# The Perioperative Neurocognitive Disorder Prediction Based on AI-Assisted EEG Dynamic Features in Anesthetized Mice

**DOI:** 10.3390/diagnostics16081186

**Published:** 2026-04-16

**Authors:** Xinyang Li, Hui Wang, Qingyuan Miao, Rui Zhou, Mengfan He, Hanxi Wan, Yuxin Zhang, Qian Zhang, Zhouxiang Li, Qianqian Wu, Zhi Tao, Xinwei Huang, Enduo Feng, Qiong Liu, Yinggang Zheng, Guangchao Zhao, Lize Xiong

**Affiliations:** 1 Shanghai Key Laboratory of Anesthesiology and Brain Functional Modulation, Translational Research Institute of Brain and Brain-like Intelligence, Clinical Research Centre for Anesthesiology and Perioperative Medicine, Department of Anesthesiology and Perioperative Medicine, Shanghai Fourth People’s Hospital, School of Medicine, Tongji University, Shanghai 200434, China; lixinyang1206@163.com (X.L.); 18045620867@163.com (Q.M.); ruizhoukepler@126.com (R.Z.); hmf0423@tongji.edu.cn (M.H.); wanhanxi@126.com (H.W.); 15693114705@163.com (Y.Z.); 2211691@tongji.edu.cn (Q.Z.); huaixu20@126.com (Z.L.); 2412008@tongji.edu.cn (Q.W.); zhitao_2001@163.com (Z.T.); huanggenetics@tongji.edu.cn (X.H.); edfeng@tongji.edu.cn (E.F.); liuqiong8001@hotmail.com (Q.L.); 2 Department of Anesthesiology and Perioperative Medicine, Xijing Hospital, The Fourth Military Medical University, Xi’an 710032, China; 17854210611@163.com; 3 Key Laboratory of Anesthesiology, Ministry of Education of China, Xi’an 710032, China; 4 Innovation Research Institute, Xijing Hospital, The Fourth Military Medical University, Xi’an 710032, China

**Keywords:** electroencephalography (EEG), perioperative neurocognitive disorder (PND), aging brain, machine learning, general anesthesia, neurophysiological biomarkers

## Abstract

**Background**: Postoperative neurocognitive disorders (PND) are frequent complications in the elderly surgical patients, with aging recognized as a major risk factor. This study aimed to identify electrophysiological markers and establish an exploratory machine learning framework for PND-related vulnerability prediction using anesthetic electroencephalography (EEG) features in aged mice. **Methods**: Young and aged mice underwent laparotomy under isoflurane anesthesia with EEG recording. Neurocognitive performance was quantified by 16 standardized behavioral fractions. A semi-supervised K-means algorithm, anchored on young-surgery mice, stratified aged-surgery mice into PND and non-PND clusters. EEG dynamics during anesthesia maintenance and emergence were analyzed, and machine learning models were trained to predict PND from EEG features. **Results**: At baseline, neurocognitive function was comparable across groups. After anesthesia/surgery, aged mice exhibited selective spatial and contextual memory impairments, with two-thirds classified as PND. During emergence, PND mice displayed elevated δ power and reduced α and β ratios. A Multi-layer Perceptron classifier showed discriminatory performance for PND classification in one evaluation setting (AUC = 0.94). **Conclusions**: This study identifies emergence-related EEG features associated with postoperative neurocognitive vulnerability in aged mice and provides an exploratory machine learning framework for preclinical risk stratification. These findings support further mechanistic investigation and warrant future validation in human perioperative EEG datasets.

## 1. Introduction

Postoperative neurocognitive disorders (PND), encompassing postoperative delirium (POD) and longer-term cognitive decline, represent a major complication among elderly surgical patients [1]. Clinically, PND presents with impairments in attention, memory, and executive function [2,3]. These deficits are associated with prolonged hospitalization, accelerated neurodegeneration, and increased mortality [4,5], creating a considerable social and economic burden [6]. Advanced age remains the predominant risk factor, with individuals older than 85 years exhibiting markedly elevated susceptibility [7,8]. However, considerable inter-individual variability in age-related vulnerability renders the early identification of high-risk individuals particularly challenging.

Aging is accompanied by synaptic loss [9], heightened neuroinflammatory susceptibility [10], and disruption of large-scale brain network connectivity [11]. These changes increase the brain’s sensitivity to anesthesia- and surgery-related stress [12]. However, the pronounced mechanistic heterogeneity underlying these vulnerabilities has hindered the development of precise, dynamic, and biologically informed risk-stratification tools [13]. Therefore, identifying stable electrophysiological markers that reflect the aging brain’s susceptibility to PND remains an urgent clinical need.

Electroencephalography (EEG) is widely used during anesthesia to monitor neural activity. It offers several advantages, including non-invasiveness, low cost, and high temporal resolution [14,15]. Previous studies have demonstrated significant EEG alterations associated with aging and cognitive impairment [13,16], as well as characteristic perioperative patterns in patients who subsequently develop PND. Nonetheless, conventional EEG indices, such as alpha power [17], spectral edge frequency [18], and burst suppression ratio [19,20], show inconsistent predictive value, likely because single-parameter metrics fail to capture the complex, nonlinear dynamics of vulnerable aging neural circuits. These limitations underscore the need for more advanced analytical strategies.

Machine learning enables the extraction of high-dimensional temporal and spectral features from EEG recordings and can detect subtle electrophysiological patterns [21,22,23]. Although machine learning has been applied to estimate anesthetic depth [22,24,25], most studies rely on single composite indices and lack behavioral or mechanistic validation, limiting their translational relevance for PND prediction [1,19,26]. Thus, establishing an animal model that enables precise correspondence between PND-related behavioral phenotypes and perioperative EEG alterations is essential.

The present study extends the existing literature in several ways. First, rather than treating aged surgical mice as a behaviorally homogeneous population, we used a behavior-informed stratification framework to capture inter-individual heterogeneity in postoperative vulnerability. Second, we incorporated peri-anesthetic EEG into an aged mouse model and focused on emergence-phase oscillatory recovery, an electrophysiological window that remains underexplored in preclinical PND research. Third, we characterized a distinct emergence EEG pattern associated with the vulnerable subgroup across the full emergence process. Fourth, we established an exploratory and interpretable machine learning framework linking peri-emergence EEG features to postoperative vulnerability phenotypes in aged mice, thereby providing a preclinical basis for future mechanistic and translational investigation of peri-emergence EEG markers.

## 2. Materials and Methods

### 2.1. Animals

All animal procedures were approved by the Ethics Committee of the Laboratory Animal Center of Tongji University and conducted in accordance with institutional guidelines for the care and use of laboratory animals. Male C57BL/6J wild-type (WT) mice were employed, comprising a young cohort (3–4 months, 30 ± 5 g) and an aged cohort (18–20 months, 42 ± 5 g), randomly assigned to experimental groups. Aged mice were obtained from Shanghai Model Organisms Center, Inc. (Shanghai, China) and Shanghai SIPPR–BK Laboratory Animal Co., Ltd. (Shanghai, China), while young mice were purchased from SPF (Beijing) Biotechnology Co., Ltd. (Beijing, China). All mice were housed in the SPF facility of the Shanghai Key Laboratory of Anesthesiology and Brain Functional Modulation, co-housed with littermates (four per cage). Housing conditions were maintained at 20–22 °C with 50 ± 10% humidity under a 12 h light/12 h dark cycle, with ad libitum access to food and water. Adhering to the 3R principles, efforts were made to minimize the number of animals and alleviate their suffering.

### 2.2. Laparotomy with EEG Monitoring Procedure

EEG rhythms were acquired from each mouse using an 8-channel EEG/electromyogram (EMG) miniature digital amplifier (Medusa, Yige Biotechnology Co., Ltd., Nanjing, China) at a sampling rate of 1000 Hz, with a band-pass filter of 0.5–70 Hz [27]. Concurrent video, EEG, and EMG signals distinguished anesthesia states. Before recording, mice were connected to the EEG device and acclimated in the recording environment for 1 h. Subsequent EEG collection included a 1 h preoperative baseline, followed by induction with 1.4% isoflurane. After 0.5 h of stabilization, a laparotomy was initiated under 1 L/min 50% oxygen flow [28]. The 0.5 h procedure involved a midline incision 1 cm below the sternum, peritoneum separation to expose abdominal contents, exteriorization of a 5 cm intestinal segment, wrapping in 0.9% saline-soaked gauze, and gentle manipulation for 10 min. The intestine was repositioned, and the peritoneum and skin were sutured separately. Incision sites were infiltrated with bupivacaine (0.25%, 3 mg/kg) for analgesia, with a heating pad ensuring warmth. Post-surgery, 1 h EEG data were recorded during anesthesia maintenance, totaling a 2 h anesthesia period [29]. The awakening phase, defined as the interval from anesthetic cessation to righting reflex recovery, was monitored, with 1 h post-anesthesia EEG data collected.

Mice rested for three days post-surgery, with autonomous activity and recovery assessed on Day 3 via a 5 min open field test. The apparatus was a non-reflective white acrylic arena (40 × 40 × 40 cm). Mouse activity was tracked by an infrared video system mounted above the arena (#68025, RWD Life Science Co., Ltd., Shenzhen, China) at 30 Hz. Mice were acclimated to the testing room for 1 h prior to assessment, and each trial began with the animal being gently placed in the center of the arena. Activity trajectories were tracked for 5 min, and the arena was cleaned with 70% ethanol post-trial to remove olfactory residues. Behavioral data were analyzed using automated software (Smart v3.0, RWD Life Science Co., Ltd., Shenzhen, China). Mice with total distances below 700 cm (young) or 500 cm (aged) were deemed excessively frail, precluding cognitive behavioral assessment, and excluded from subsequent experiments.

### 2.3. Cognitive Function Assessment

#### 2.3.1. Barnes Maze Test

The Barnes maze was employed to assess spatial reference memory and learning ability in mice [30]. The apparatus comprised a circular platform (120 cm in diameter) positioned 100 cm above the floor, containing 20 equally spaced peripheral holes (5 cm in diameter), of which only one led to a dark escape box. Bright overhead lighting (>1000 lux), continuous background noise (80–90 dB), and fixed distal visual cues served as motivational and spatial references.

The experiment comprised habituation, acquisition, and probe phases. On postoperative Day 3, mice were habituated by observing visual cues for 3 min and exploring the target hole for 1 min before being guided into the escape box. During acquisition, each mouse completed 15 trials over four days (180 s limit, ≥30 min inter-trial interval). Mice failing to locate the escape hole were guided into it for 1 min. The probe test conducted 24 h later without the escape box. Mice were allowed to explore freely for 120 s, during which the latency to first visit the target hole, the number of visits, and the proportion of time spent in the target quadrant were quantified as indices of spatial memory retention.

#### 2.3.2. Contextual Fear Conditioning Test

The contextual fear conditioning test consisted of habituation, acquisition, and testing phases. Habituation was performed on postoperative Day 8. Mice were placed individually into a white plastic conditioning chamber (21 × 21 × 26 cm) and allowed to explore freely for 2 min without stimulation. After 24 h, the acquisition phase was conducted. Following 2 min of free exploration, mice received five foot-shock trials. Each trial lasted 35 s, consisting of 33 s of free exploration followed by a 0.7 mA foot shock for 2 s. Twenty seconds after the final shock, mice were returned to their home cages.

Contextual memory was assessed 24 h later. Mice were reintroduced into the same chamber, with all contextual features preserved, and allowed to explore for 5 min without shock. Freezing behavior, defined as the absence of movement except for respiration, was quantified as an indicator of memory for the association between the training context and the aversive foot shock. Mice that successfully encoded the association typically exhibited increased freezing duration and a greater number of freezing bouts. The chamber was cleaned thoroughly with 70% ethanol between sessions to eliminate olfactory cues.

### 2.4. Feature Dimensionality Reduction

To visualize the structure of the 16 standardized behavioral fractions, dimensionality reduction was applied to transform the high-dimensional dataset into an interpretable space. Principal component analysis (PCA) was used to project behavioral profiles from young and aged mice, with or without surgery, onto a two-dimensional coordinate system [31]. For a dataset $X\in R^{n\times d}$ with *n* samples and *d* features, PCA computes a covariance matrix $C=\frac{1}{n-1}X^{T}X$ (assuming centered data). Eigenvalue decomposition is performed on *C* to obtain eigenvalues $\lambda_{i}$ and eigenvectors $\nu_{i}$. The eigenvectors associated with the largest eigenvalues form the principal components, defining a new coordinate system. The data is projected onto these components to produce a reduced representation $Y=XV_{k}$, which $V_{k}$ contains the top *k* eigenvectors (e.g., *k* = 2 for two-dimensional output).

### 2.5. Wasserstein Distance

To evaluate group separation in the two-dimensional PCA space, the Wasserstein distance (WD, Earth Mover’s Distance) was computed between groupwise multivariate normal distributions [32]. For each group, PCA coordinates were modeled using a mean vector μ and covariance matrix Σ. The mean vector $\mu_{i}$ for group *i* was calculated as the sample mean of the PCA coordinates, and the covariance matrix $\Sigma_{i}$ was computed using the sample covariance of the same coordinates. The WD between two groups with distributions $N\left(\mu_{1},\Sigma_{1}\right)$ and $N\left(\mu_{2},\Sigma_{2}\right)$ was calculated using the function:

(1) $$W_{\left(N\left(\mu_{1},\Sigma_{1}\right),N\left(\mu_{2},\Sigma_{2}\right)\right)}=\sqrt{||\mu_{1}-\mu_{2}|{|}^{2}+||\sqrt{\Sigma_{1}}-\sqrt{\Sigma_{2}}|{|}_{F}^{2}}$$

Here, $∥\mu_{1}-\mu_{2}{∥}^{2}$ represents the squared Euclidean distance between the mean vectors, capturing the difference in the centers of the distributions. The term $||\sqrt{\Sigma_{1}}-\sqrt{\Sigma_{2}}|{|}_{F}^{2}$ denotes the squared Frobenius norm of the difference between the matrix square roots of the covariance matrices, reflecting differences in the shapes of the distributions. The matrix square root $\sqrt{\Sigma_{i}}$ was computed using numerical methods (e.g., eigenvalue decomposition), ensuring $\sqrt{\Sigma_{i}}\cdot \sqrt{\Sigma_{i}}=\Sigma_{i}$. The resulting values were used to quantify the degree of separation between groups, with larger distances indicating greater separation in the PCA space.

### 2.6. PND Identification Based on a Semi-Supervised Learning Algorithm

In this study, semi-supervised constraint-based clustering was applied to incorporate partial label information into the K-means framework, thereby enabling biologically meaningful subgrouping [33].

Standard K-means partitions data into *K* clusters by minimizing the within-cluster sum of squares (WCSS), expressed as:

(2) $$J=\sum_{i=1}^{K}\sum_{x_{j}\in C_{i}}||x_{j}-\mu_{i}|{|}^{2}$$

where $C_{i}$ is the set of data points in cluster *i*, $\mu_{i}$ is the centroid of cluster *i*, $x_{i}$ are the data points, and $\|\cdot {\|}^{2}$ denotes the square of Euclidean distance.

To incorporate prior information, “must-link’’ constraints were imposed such that a predefined subset of samples was forced into the same cluster. To enforce these constraints, we modified the standard K-means procedure using custom initialization.

The number of clusters was set to *K* = 2 because the primary objective of this study was not to discover an unrestricted number of behavioral subtypes, but to operationalize the aged postoperative cohort into two biologically interpretable groups relative to the young-surgery reference: a behaviorally preserved/non-PND-like subgroup and a vulnerable/PND-like subgroup. This choice should therefore be interpreted as a hypothesis-guided stratification framework rather than evidence that postoperative neurocognitive vulnerability is intrinsically binary. The rationale for retaining the K-means framework, as well as its comparison with alternative unconstrained clustering methods [34], is provided in the Supplementary Information, together with an evaluation of clustering stability across random seeds using the Adjusted Rand Index (ARI) [35].

The procedure involved the following steps:

Data Preparation: Sample IDs and feature values were extracted from the input dataset.

Constraint Identification: The indices of the constrained samples were identified.

Centroid Initialization for Constraints: The mean of the fixed samples as one initial centroid were computed to bias them toward the same cluster:

(3) $$\mu_{1}=\frac{1}{m}\sum_{j=1}^{m}Xf_{j}$$

where *m* is the number of constrained samples, and $Xf_{j}$ are their feature vectors. Randomly select a point from the non-fixed samples as the second initial centroid $\mu_{2}$.

K-means execution: After specifying *K* = 2, constrained K-means clustering was performed using standard assignment and update steps under the imposed semi-supervised constraints.

Assignment step: For each data point $x_{j}$,

(4) $$c_{j}=arg\underset{i}{\min}{\left||x_{j}-\mu_{i}|\right|}^{2}$$

In the update step, each cluster centroid was recalculated based on the samples assigned to that cluster:

Update step: For each cluster *i*,

(5) $$\mu_{i}=\frac{1}{C_{i}}\sum_{x_{j}\in C_{i}}x_{j}$$

To improve reproducibility, clustering was performed using a fixed random seed and a single initialization. This framework combines data-driven clustering with biologically informed guidance and was considered suitable for the present dataset, in which partial prior knowledge was available from the young-surgery reference group.

### 2.7. EEG Signal Analysis

EEG recordings were processed to derive quantitative electrophysiological features, including burst suppression ratio (BSR), edge frequency, and power spectral density (PSD).

All analyses were performed using Python 3.10.13 with libraries including NumPy 1.26.4, SciPy 1.15.2, and Matplotlib 3.8.4. Data were sampled at 1000 Hz and pre-filtered with a 4th-order Butterworth band-pass filter (1–48 Hz) to attenuate noise and movement-related artifacts [36].

#### 2.7.1. Burst Suppression Ratio

BSR was computed to quantify the proportion of time the EEG remained in a suppressed, low-amplitude state. Consistent with commonly used criteria in the burst suppression literature, suppression epochs were defined as periods in which the EEG amplitude remained within ±5 μV for at least 0.5 s [37,38,39]. The minimum duration requirement was used to reduce misclassification of brief low-amplitude fluctuations or noise as true suppression. Suppression detection was performed by identifying consecutive samples meeting the amplitude criterion and accumulating the total number of samples in valid suppression epochs. The BSR was then calculated as:

(6) $$Band_{Percentage}=\left(\frac{Total_{suppressionsamples}}{Total_{signalsamples}}\right)\times 100\%$$

This approach ensures that transient low-amplitude fluctuations are not misclassified as suppression. In our analysis, BSR values were computed for individual EEG segments, providing insights into anesthetic depth variations.

#### 2.7.2. Edge Frequency

The edge frequency, defined as the frequency below which 95% of total spectral power is contained [40], was obtained using short-time Fourier transform (STFT). Spectrograms were computed using a 1 s Hamming window with 75% overlap. For each time slice, cumulative spectral power was integrated from low to high frequencies, and the 95% cutoff point was recorded as the edge frequency.

#### 2.7.3. Power Spectral Density

PSD was estimated with Welch’s method (2 s Hann window, 50% overlap), yielding power spectra expressed in µV^2^/Hz. PSD analysis enabled quantification of dominant oscillatory bands and facilitated comparison of frequency-domain alterations during anesthesia emergence [40].

### 2.8. Machine Learning Prediction Model Development

#### 2.8.1. Feature Extraction and Dataset Definition

EEG signals recorded during the emergence period were processed to derive a comprehensive set of 33 predefined features, capturing both time-domain and frequency-domain characteristics. Signals sampled at 1000 Hz were filtered with a 4th-order Butterworth band-pass filter (1–48 Hz) and segmented into non-overlapping 2 s epochs. For each epoch, 33 distinct features were computed, and the resulting values were then averaged across epochs to obtain a single feature vector for each animal. The extracted features, detailed in Table S2, included band-limited power, statistical descriptors, entropy-related metrics, and Hjorth parameters. This predefined EEG feature set was assembled a priori based on commonly used handcrafted EEG descriptors reported in prior EEG-analysis studies [41], and implementation of selected descriptors was also informed by a publicly available EEG feature-extraction toolbox (JingweiToo, *EEG-Feature-Extraction-Toolbox*, GitHub, repository, available online: https://github.com/JingweiToo/EEG-Feature-Extraction-Toolbox, accessed on 1 April 2026).

For machine learning analysis, a total of 38 mice were included. The non-PND class comprised 18 animals, including 8 young mice and 10 aged mice classified as non-PND, whereas the PND class comprised 20 aged mice classified as PND. The PND/non-PND labels were defined according to the PCA-based behavioral stratification described above. Young mice were incorporated into the non-PND class as a behaviorally preserved reference group. Accordingly, the classifier should be interpreted as distinguishing aged-PND-vulnerable mice from a preserved/non-PND-like reference class, rather than as a strictly age-matched aged non-PND versus aged-PND classifier.

#### 2.8.2. Preprocessing, Training, and Validation Workflow

No separate feature-selection or dimensionality-reduction step was applied in the primary machine learning analysis; all 33 predefined EEG features were entered into each classifier. Feature scaling was performed using StandardScaler within a scikit-learn Pipeline (scikit-learn 1.6.1), such that scaling parameters were fitted exclusively on the training data in each cross-validation loop, thereby preventing information leakage from validation or test data.

The full dataset was evaluated under three train:test ratios (6:4, 7:3, and 8:2) to assess the dependence of model performance on data partitioning. For each split ratio, evaluation was repeated using four random seeds (42, 300, 600, and 800). Within each training set, model development and hyperparameter tuning were performed using StratifiedKFold cross-validation (n_splits = 3) and GridSearchCV, so that class proportions were preserved across folds. After hyperparameter optimization, the final model was retrained on the full training set and evaluated once on the untouched test set. The held-out test set was not used in any preprocessing, model selection, or hyperparameter optimization step.

#### 2.8.3. Machine Learning Models and Evaluation Metrics

Twelve supervised classifiers were evaluated: Logistic Regression (LR) [42], CatBoost [43], Decision Tree (DT) [44], Random Forest (RF) [45], AdaBoost [46], Extra Trees (ET) [47], Support Vector Machine (SVM) [48], Gaussian Naive Bayes (NB) [49], K-Nearest Neighbors (KNN) [50], Multi-layer Perceptron (MLP) [51], eXtreme Gradient Boosting (XGBoost) [52], and Light Gradient Boosting Machine (LightGBM) [53]. A detailed description of each algorithm is provided in the Supplementary Materials.

Model performance was assessed using five metrics: accuracy, precision, recall, F1-score, and the area under the receiver operating characteristic curve (AUC) [54,55]. The definitions of these metrics are provided below, where *TP*, *TN*, *FP*, and *FN* denote true positives, true negatives, false positives, and false negatives, respectively. Because the present task was a binary classification problem with a limited sample size and slight class imbalance, AUC was used as the primary discrimination metric, while the remaining metrics were used as complementary indicators of classification behavior. To further evaluate robustness, repeated-resampling comparisons across different train:test ratios and random seeds were additionally summarized in the Supplementary Materials.

Accuracy: Measures the proportion of correctly classified samples.

(7) $$Accuracy=\frac{Number_{CorrectPredictions}}{TotalNumber_{Predictions}}=\frac{TP+TN}{TP+TN+FP+FN}$$

Precision: Quantifies the proportion of predicted positive samples that are correct.

(8) $$Preci\sin=\frac{TP}{TP+FP}$$

Recall: Measures the proportion of actual positive samples correctly identified.

(9) $$Recall=\frac{TP}{TP+FN}$$

F1 Score: The harmonic mean of Precision and Recall, balancing both metrics.

(10) $$F1Score=2\cdot \frac{Precision\cdot Recall}{Precision+Recall}$$

AUC (Area under the ROC curve): Evaluates the model’s ability to distinguish between classes by plotting the true positive rate (recall) against the false positive rate (*FP*/(*FP* + *TN*)) across various thresholds. AUC ranges from 0 to 1, with 1 indicating perfect discrimination. Computed numerically via the trapezoidal rule over the ROC curve.

#### 2.8.4. Model Explanation

Model explainability was achieved through SHAP analysis, which assigns additive contribution values to each feature based on cooperative game theory [56,57]. Mean absolute SHAP values ranked the relative global importance of predictors, whereas sample-level SHAP distributions (bee-swarm plots) illustrated both the direction and magnitude of feature effects. These visualizations enabled transparent interpretation of the classifier’s decision process.

### 2.9. Statistical Analysis

All statistical procedures were performed in Prism 9 (GraphPad Software, San Diego, CA, USA). Data are presented as mean ± SEM, and sample sizes are specified in the corresponding figure legends. Distributional properties of each dataset were first evaluated using the Shapiro–Wilk test, and variance equality was examined with Levene’s test prior to groupwise comparisons.

For two-group analyses, normally distributed data with equal variances were examined using two-tailed unpaired Student’s *t* tests; when variances differed, Welch’s correction was applied. Non-parametric data were assessed using the Mann–Whitney U test. For single-factor comparisons involving three groups, one-way ANOVA with Tukey’s post hoc test was used when parametric assumptions were met; Welch’s ANOVA followed by the Games–Howell test was applied when variances were unequal; and the Kruskal–Wallis test with Dunn’s post hoc procedure was used for non-parametric data. Multi-factor datasets were analyzed using two-way ANOVA with Sidak-adjusted post hoc comparisons. Statistical significance was defined as *p* < 0.05. Significance notations were as follows: n.s. (no significant), *p* > 0.05; * *p* < 0.05; ** *p* < 0.01; *** *p* < 0.001; **** *p* < 0.0001.

## 3. Results

### 3.1. Assessment of Age and Anesthesia/Surgery Effects on Cognitive Behavior

**Figure 1:**
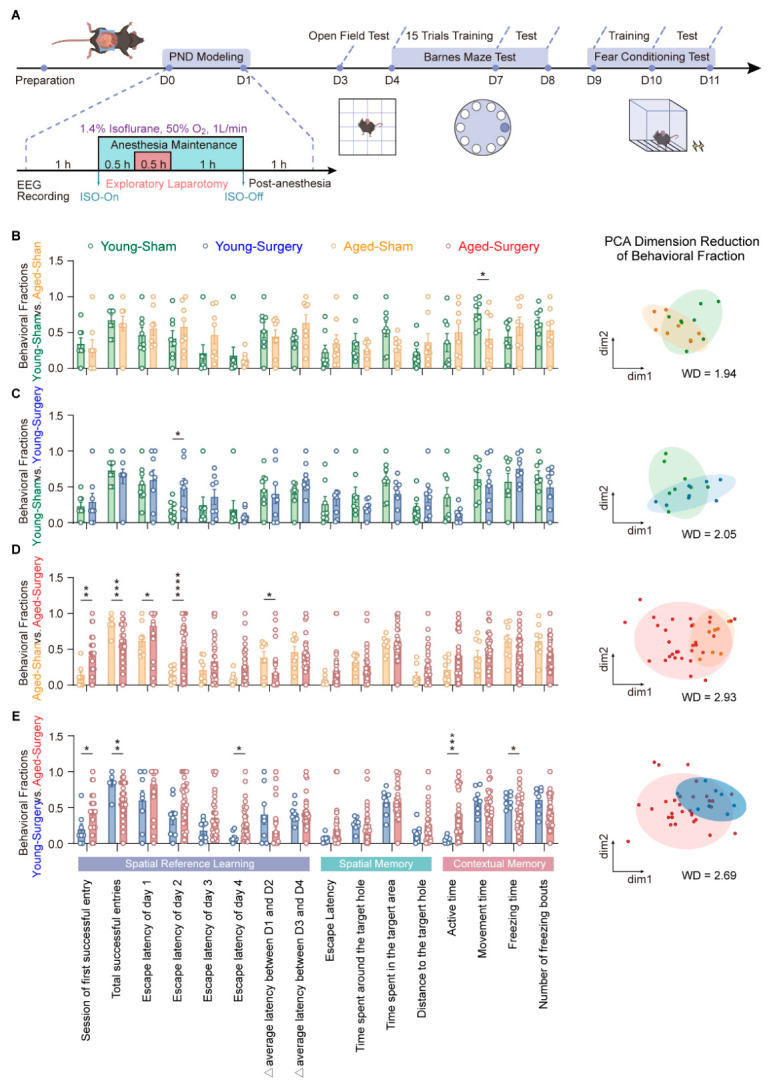
Age- and surgery-related alterations in cognition-associated behavioral performance. (**A**) Schematic diagram of the experimental workflow. (**B**) Comparison between young-sham and aged-sham groups across 16 standardized behavioral fractions. The two groups exhibited largely similar performance in BMT and FCT, except for reduced freezing time in young mice during FCT (young-sham, *n* = 8; aged-sham, *n* = 8, unpaired *t* test, df = 14, F [7, 7] = 3.271; *p* = 0.0262). Right: 2D PCA embedding of the two groups. (**C**) Comparison between the young-sham and young-surgery groups. Young-surgery mice exhibited prolonged escape latency on Day 2 of Barnes maze training, while overall learning efficiency and contextual memory remained intact (young-sham, *n* = 8; young-surgery, *n* = 8, unpaired *t* test with Welch’s correction, df = 8.949, F [7, 7] = 7.042; *p* = 0.0395). Right: 2D PCA embedding of the two groups. (**D**) Comparison between the aged-sham and aged-surgery groups. Aged-surgery mice displayed significant spatial learning impairments, including prolonged escape latency, delayed first hole latency, and reduced total successful entries (aged-sham, *n* = 8; aged-surgery, *n* = 30; session of first successful entry: Mann–Whitney test, *U* = 41.50, *p* = 0.0030; total successful entries: unpaired *t* test with Welch’s correction, df = 25.45, F [29, 7] = 4.598, *p* = 0.0002; escape latency of Day 1: Mann–Whitney test, *U* = 60, *p* = 0.0209; escape latency of Day 2: unpaired *t* test with Welch’s correction, df = 33.83, F [29, 7] = 8.977, *p* < 0.0001; △ average latency between D1 and D2: Mann–Whitney test, *U* = 60, *p* = 0.0211). Contextual fear memory fractions, however, did not differ between the two groups. Right: 2D PCA embedding of the two groups. (**E**) Comparison between the young-surgery and aged-surgery groups. Aging significantly exacerbated postoperative impairments in both spatial learning and contextual memory (young-surgery, *n* = 8; aged-surgery, *n* = 30; session of first successful entry: Mann–Whitney test, *U* = 61.50, *p* = 0.0327; total successful entries: Mann–Whitney test, *U* = 48.50, *p* = 0.0078; escape latency of Day 4: Mann–Whitney test, *U* = 53, *p* = 0.0147; high-active time: Mann–Whitney test, *U* = 22, *p* = 0.0001; freezing time: unpaired *t* test with Welch’s correction, df = 31.29, F [29, 7] = 7.027, *p* = 0.0102). Right: 2D PCA embedding of the two groups. All data are presented as mean ± SEM, * *p* < 0.05, ** *p* < 0.01, *** *p* < 0.001, **** *p* < 0.0001, with independent group comparisons corrected for multiple testing.

To systematically evaluate the impact of age and anesthesia/surgery on cognitive behaviors, laparotomy under isoflurane anesthesia was performed in both young and aged mice with simultaneous EEG monitoring. After a three-day recovery period, all mice underwent an open field test (OFT) to assess baseline locomotor capacity. Cognitive phenotypes were further quantified across 16 standardized behavioral fractions using the Barnes maze test (BMT) and contextual fear conditioning test (CFCT). Detailed protocols for EEG recording, anesthesia/surgery, and behavioral assessments are shown in Figure 1A.

To assess age-related effects at baseline, we first compared the 16 behavioral fractions across four groups: young-sham, aged-sham, young-surgery, and aged-surgery (Figure 1B–E). As shown in Figure 1B, young-sham and aged-sham mice exhibited minimal differences in 15 of 16 fractions, with a Wasserstein distance (WD) of 1.94, indicating largely comparable cognitive performance in the absence of surgery. The only significant age difference was observed in CFCT freezing measures, where young mice showed shorter freezing time than aged mice, consistent with higher baseline activity in younger animals. Overall, these findings indicate broadly similar baseline spatial learning and contextual memory retrieval between age groups, apart from a modest age-related difference in freezing behavior.

Likewise, no major separation was detected between young-sham and young-surgery groups (WD = 2.05; Figure 1C). Young mice showed only a mild prolongation of escape latency on Day 2 of BMT training, whereas overall learning efficiency and spatial memory retrieval remained preserved, suggesting robust resilience to anesthesia/surgery stress. In contrast, aged mice subjected to surgery displayed pronounced impairments in spatial reference memory acquisition, with a markedly larger WD of 2.93 between aged-sham and aged-surgery groups (Figure 1D). Aged-surgery mice exhibited prolonged escape latencies on Days 1–2, an increased Δ average latency between Day 1 and Day 2, delayed sessions of first successful entry, and reduced total successful entries. Although final memory retrieval performance did not differ significantly from sham controls, the learning curve was substantially flattened, supporting increased brain vulnerability with aging and indicating that age is a major contributor to PND-like susceptibility.

We therefore selected the young-surgery group as the primary reference to examine age-dependent postoperative alterations. As shown in Figure 1E, the WD between young-surgery and aged-surgery groups reached 2.69, reflecting substantial multivariate separation. Aged-surgery mice showed fewer total successful entries, delayed first successful entry, prolonged escape latency on Day 4, reduced freezing time, and increased high-active time. These results indicate that, despite broadly comparable baseline cognition, aged brains develop deficits in anterograde spatial memory acquisition and contextual fear memory after anesthesia/surgery. This pattern is consistent with the idea that PND susceptibility in aged individuals arises from age-related neural fragility and reduced network plasticity, rather than perioperative stress exposure alone.

### 3.2. Cluster Analysis of Standardized Behavioral Fractions Distinguishes PND Manifestations in Aged-Surgery Mice

**Figure 2:**
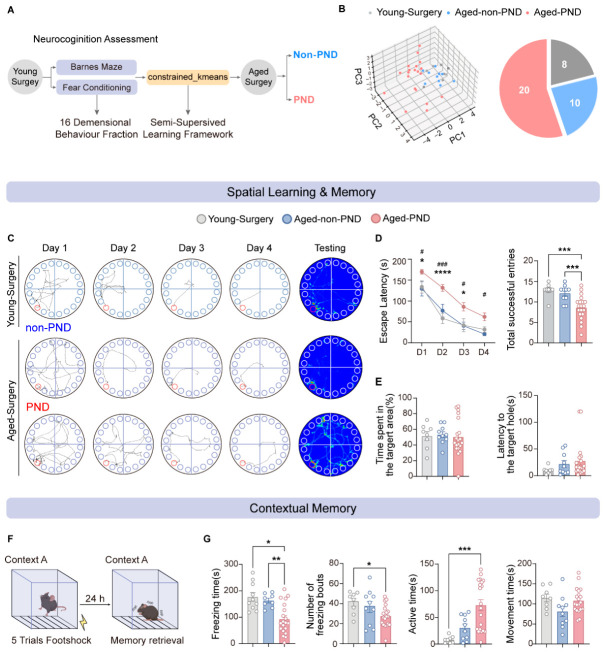
Identification of PND subgroups by clustering and behavioral assessments. (**A**) Schematic of the K-means clustering workflow based on 16 standardized behavioral fractions. (**B**) Low-dimensional representation of individual behavioral data. Left: 3D PCA embedding of young-surgery (grey, *n* = 8), aged non-PND (blue, *n* = 10), and aged-PND (red, *n* = 20) mice. Thirty-eight samples were reduced from 16-dimensional behavioral fractions into 3D space and separated by K-means clustering. Right: pie chart illustrating the proportion of each subgroup. (**C**) Representative trajectories during Barnes maze training (Days 1–4) and probe test (Day 5) for young-surgery, aged-non-PND, and aged-PND mice. (**D**) Barnes maze training performance. Left: Escape latency across four training days. Aged-PND mice showed significantly prolonged escape latencies compared with young-surgery mice (significance between young-surgery and aged-PND groups is denoted by #, where # *p* < 0.05 and ### *p* < 0.001, two-way ANOVA, with Tukey’s multiple comparisons post hoc test, F [2, 140] = 32.47, *p* < 0.0001; Day 1, Adjusted *p* Value = 0.0196; Day 2, Adjusted *p* Value = 0.0006; Day 3, Adjusted *p* Value = 0.0128; Day 4, Adjusted *p* Value = 0.0199) and with aged-non-PND mice on training Days 1–3 (significance between aged-non-PND and aged-PND groups is denoted by *, Day 1, Adjusted *p* Value = 0.0204; Day 2, Adjusted *p* Value < 0.0001; Day 3, Adjusted *p* Value = 0.0159). Right: Total successful entries across 15 training trials, showing reduced entries in aged-PND compared with both young-surgery and aged-non-PND mice (Kruskal–Wallis with Dunn’s post hoc tests, H [df = 2] = 18.76, *p* < 0.0001; young-surgery vs. PND, Adjusted *p* = 0.0012; non-PND vs. PND, Adjusted *p* = 0.0016; young-surgery, *n* = 8; aged-non-PND, *n* = 10; aged-PND, *n* = 20). (**E**) Probe test results. Left: Percentage of time spent in the target quadrant (Welch’s ANOVA with Games–Howell post hoc tests, W [2.000, 18.61] = 0.005568, *p* = 0.9944). Right: Latency to first reach the target hole (Kruskal–Wallis with Dunn’s post hoc tests, H [df = 2] = 3.147, *p* = 0.2073); no significant differences were observed among the three groups. (**F**) Schematic of the contextual fear conditioning paradigm. Mice received five foot shocks during training, followed by contextual testing 24 h later. Freezing behavior was measured as an index of contextual memory. (**G**) Contextual fear memory performance. Left: Freezing time, with aged-PND mice showing reduced freezing compared with young-surgery and aged-non-PND groups (Welch’s ANOVA with Games–Howell post hoc tests, W [2.000, 21.37] = 6.459, *p* = 0.0064; young-surgery vs. PND: Adjusted *p* = 0.0050; non-PND vs. PND: Adjusted *p* = 0.0155). Left-center: Number of freezing bouts, reduced in aged-PND vs. young-surgery (one-way ANOVA with Tukey’s multiple comparisons tests, F [2, 35] = 3.863, df = 2, *p* = 0.0305; young-surgery vs. PND: Adjusted *p* = 0.0395). Right-center: Active time, elevated in aged-PND vs. young-surgery (Kruskal–Wallis with Dunn’s post hoc tests, H [df = 2] = 13.69, *p* = 0.0011; young-surgery vs. PND: Adjusted *p* = 0.0006). Right: Movement time, with no significant difference among groups (one-way ANOVA with Tukey’s multiple comparisons tests, F [2, 35] = 1.412, df = 2, *p* = 0.2572). All data are presented as mean ± SEM. * *p* < 0.05, ** *p* < 0.01, *** *p* < 0.001, **** *p* < 0.0001.

To resolve inter-individual variability in PND susceptibility within the aged surgical cohort and to link these behavioral differences to EEG dynamics during anesthesia, 16 standardized behavioral fractions were subjected to clustering. A semi-supervised K-means algorithm was implemented using the young-surgery group as a non-PND reference centroid (Figure 2A). Aged-surgery mice were thereby partitioned into two clusters: an aged non-PND group (proximal to young-surgery in feature space) and an aged-PND group (more distant from young-surgery). Principal component analysis (PCA) visualization supported this separation, yielding a non-PND:PND ratio of approximately 1:2 (Figure 2B), supporting the utility of this clustering framework for identifying a sizeable vulnerable/PND-like subgroup within the aged surgical cohort.

Based on this clustering, we next compared cognitive profiles among young-surgery, aged non-PND, and aged-PND mice. During the Barnes maze training phase, aged-PND mice showed marked spatial learning deficits relative to both young and aged non-PND mice. Specifically, compared with young mice, aged-PND mice exhibited significantly prolonged escape latencies across Days 1–4; compared with aged non-PND mice, escape latencies were also significantly increased on Days 1–3. Across the entire learning process, total successful entries were reduced in PND mice relative to both young and non-PND groups (Figure 2C,D). In contrast, aged non-PND mice performed similarly to young-surgery mice, indicating preserved spatial learning capacity. In the probe trial, no significant differences were detected among groups in time spent in the target area or latency to the target hole (Figure 2C,E), suggesting that PND-related abnormalities are predominantly confined to acquisition, with spatial memory retrieval largely intact.

In the contextual fear conditioning test (Figure 2F), aged-PND mice exhibited a pronounced reduction in freezing time compared with both young and aged non-PND mice (Figure 2G). Relative to young mice, PND mice also showed fewer freezing bouts and increased high-active time. By contrast, aged non-PND mice displayed freezing and activity measures at intermediate levels, without significant differences from either young or PND mice, and movement time did not differ significantly across groups. Together, these findings delineate distinct cognitive behavioral signatures in aged-PND versus non-PND mice: only PND mice exhibit combined impairments in spatial learning and contextual fear memory, whereas non-PND mice retain cognitive resilience comparable to young controls. This pattern supports the biological relevance of our K-means-based classification in identifying PND-like manifestations in aged-surgery mice.

### 3.3. Differential Intracranial EEG Dynamics During Anesthesia and Emergence Underlying Age-Related PND Susceptibility

**Figure 3:**
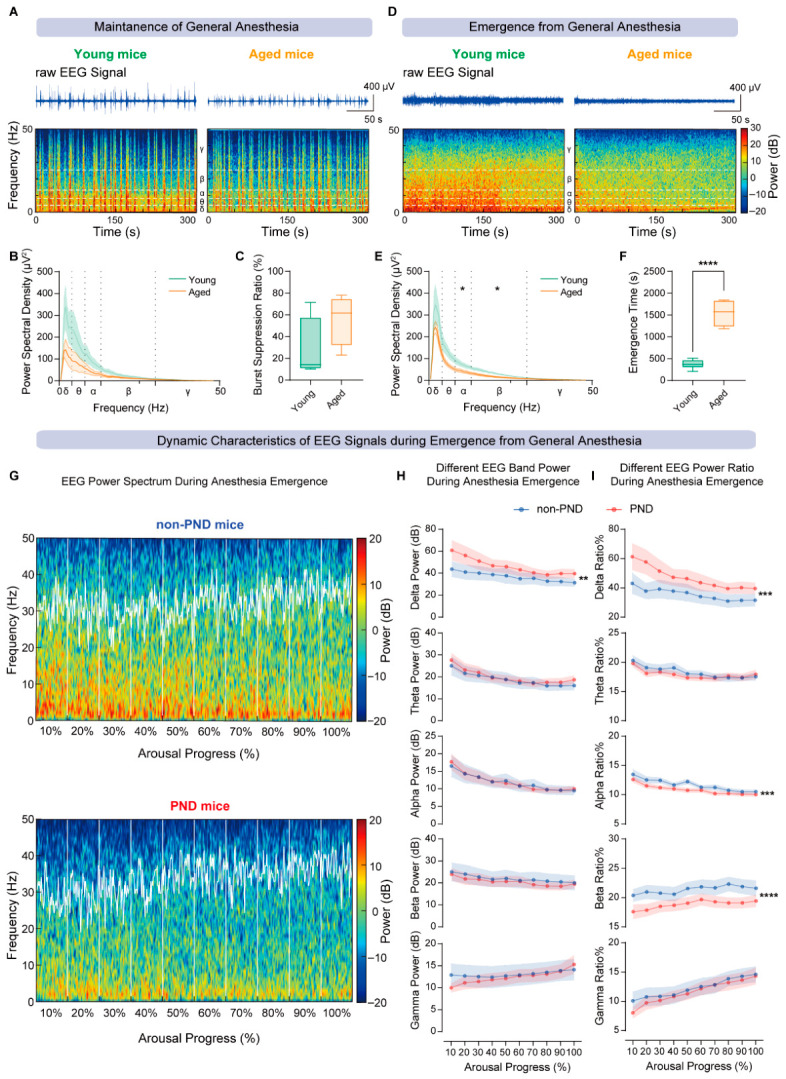
Age-related differences in EEG dynamics during anesthesia maintenance and emergence. (**A**) Representative raw EEG traces and corresponding spectrograms during the anesthesia maintenance phase in young (**left**) and aged (**right**) mice. (**B**) Comparison of power spectral density (PSD) across frequency bands (≤50 Hz) during anesthesia maintenance in young and aged mice, showing no significant differences between groups (unpaired *t* test). (**C**) Comparison of burst suppression ratio (BSR) during anesthesia maintenance, with no significant difference between young and aged mice (Mann–Whitney test). (**D**) Representative raw EEG traces and spectrograms during the emergence phase in young (**left**) and aged (**right**) mice. (**E**) Comparison of PSD across frequency bands (≤50 Hz) during emergence, showing significantly reduced α- and β-band power in aged mice relative to young mice (unpaired *t* test, df = 12; α: t = 2.247; *p* = 0.0442; β: t = 1.711; *p* = 0.0130). (**F**) Comparison of emergence time between young and aged mice, with aged mice exhibiting significantly prolonged emergence (unpaired *t* test with Welch’s correction, df = 7.709; t = 10.65; *p* < 0.0001). (**G**) Group-averaged spectrograms of EEG dynamics during the 0–100% emergence process in non-PND (**top**) and PND (**bottom**) mice, with the white trace indicating the spectral edge frequency. For each animal, the emergence period was divided into 10 equal bins spanning relative arousal progression from 0% to 100% (displayed as 10%, 20%, …, 100%). A fixed 15 s EEG segment was extracted from each bin, and spectral data from the same bin were then averaged within each group to generate the heatmaps. Both heatmaps were displayed using the same global color scale (−20 to +20 dB), with no per-animal or per-group normalization applied. (**H**,**I**) Comparison of EEG band power (γ/α/β/θ/δ) spectral density (**H**) and relative power ratios (**I**) during emergence in non-PND and PND mice. PND mice exhibited reduced α- and β-band power ratios (two-way ANOVA, df = 1; α power ratio: F [1, 280] = 15.02, *p* = 0.0001; β power ratio: F [1, 280] = 21.78, *p* < 0.0001) and increased δ-band power spectral density and ratios compared with non-PND mice (two-way ANOVA, df = 1; δ power: F [1, 280] = 9.264; *p* = 0.0026; δ power ratio: F [1, 280] = 13.64, *p* = 0.0003). non-PND, *n* = 10; PND, *n* = 20. All data are presented as mean ± SEM. * *p* < 0.05, ** *p* < 0.01, *** *p* < 0.001, **** *p* < 0.0001.

To further elucidate the neurophysiological basis of age-related PND vulnerability, intracranial EEG dynamics were first examined in young and aged mice during anesthesia maintenance and emergence. During the maintenance period, both age groups displayed predominantly low-frequency EEG activity (Figure 3A), with no significant differences in power spectral density below 50 Hz (Figure 3B) or in burst suppression ratio (Figure 3C). These findings indicate a comparable pattern of neural oscillations and a similarly deep, suppressed brain state under steady isoflurane anesthesia, irrespective of age.

By contrast, the emergence phase revealed clearly divergent recovery trajectories of EEG spectra (Figure 3D). Aged mice showed markedly reduced α (Figure 3E) and β power, accompanied by prolonged emergence times (Figure 3F), suggesting an age-related fragility in the early re-engagement of large-scale neural networks after anesthesia. Consistent with this pattern, emergence-phase EEG showed greater discriminatory value than maintenance-phase activity for subsequent subgroup characterization in the present dataset.

We then focused on differences between PND and non-PND subgroups within the aged cohort to characterize the dynamic EEG recovery process during emergence. Heatmap analysis demonstrated that, across the 0–100% arousal continuum, non-PND mice exhibited a gradual reconstitution of spectral boundaries, whereas PND mice showed delayed spectral reorganization and impaired temporal restoration of neural activity (Figure 3G). Subsequent spectral analysis revealed that PND mice had consistently higher δ power throughout emergence, with an increased δ power ratio and reduced α and β power ratios relative to non-PND mice (Figure 3H,I), indicating a persistent dominance of slow-wave activity with blunted high-frequency recovery.

Taken together, EEG activity during anesthesia maintenance was largely comparable between young and aged mice, whereas aged mice, particularly those developing PND, displayed pronounced dynamic abnormalities during emergence. These observations suggest that disrupted emergence-phase neural dynamics, characterized by sustained δ predominance and attenuated α and β restoration, represent a critical electrophysiological correlate of postoperative neurocognitive vulnerability in aged mice and a neurophysiologically informative window for future mechanistic and translational investigation.

### 3.4. Machine Learning Model for Predicting PND vs. Non-PND

Building on the analysis of emergence-phase EEG dynamics in aged-PND and non-PND mice, 33 quantitative EEG features were extracted from the emergence period (definitions in Table S2). A direct comparison of these features revealed no significant group differences between PND and non-PND mice (Figure S1), indicating that simple averages of emergence EEG features are insufficient to distinguish PND susceptibility. We therefore applied machine learning approaches to predict PND occurrence in aged mice based on multidimensional EEG features during emergence. For machine learning analysis, a total of 38 mice were included, comprising 18 animals in the non-PND class (8 young mice and 10 aged non-PND mice) and 20 animals in the PND class (20 aged-PND mice). The PND/non-PND labels were defined according to the PCA-based behavioral stratification described above. Young mice were incorporated into the non-PND class as a behaviorally preserved reference group. Accordingly, the classifier should be interpreted as distinguishing aged-PND-vulnerable mice from a preserved/non-PND-like reference class, rather than as a strictly age-matched aged non-PND versus aged-PND classifier. Twelve algorithms, including Logistic Regression, CatBoost, Decision Tree, Random Forest, AdaBoost, Extra Trees, Support Vector Machine, Gaussian Naive Bayes, K-Nearest Neighbors, Multi-layer Perceptron (MLP), XGBoost, and LightGBM, were evaluated for PND versus non-PND classification.

**Figure 4:**
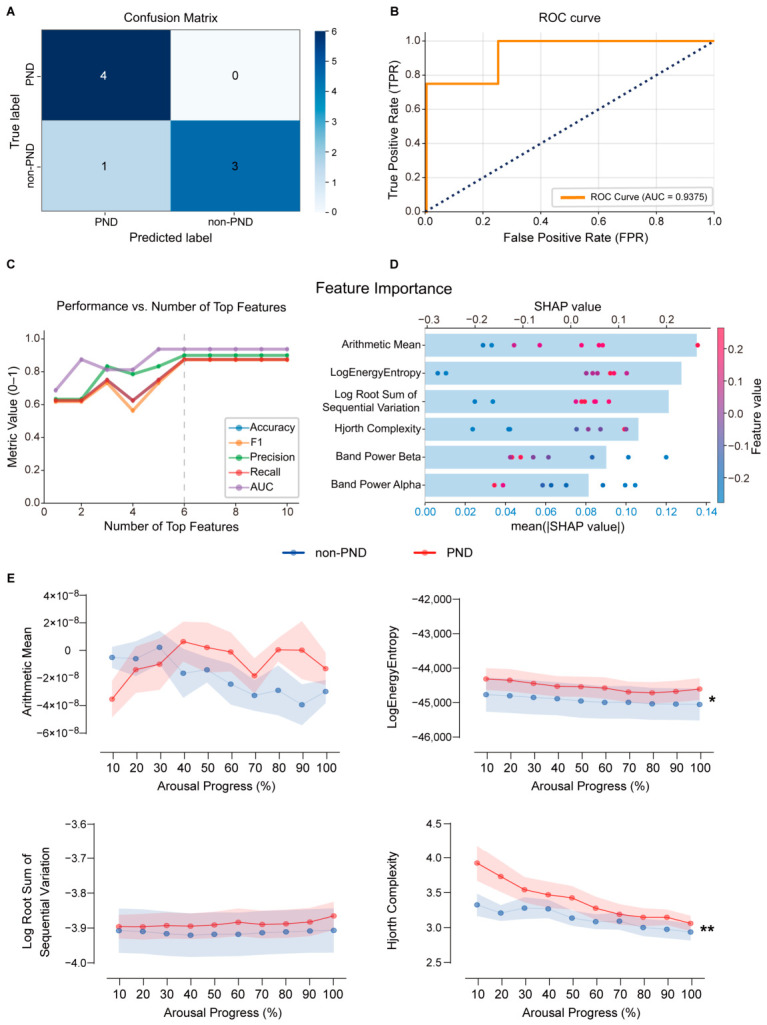
Performance of MLP model predicting PND vs. non-PND in the test set. (**A**) The confusion matrix. (**B**) Receiver operating characteristic (ROC) curve analysis (the dotted diagonal line indicates the chance level). (**C**) Performance as a function of the number of top features; the vertical dotted line indicates the inclusion of the top six features, after which model performance reached a plateau. (**D**) Importance ranking plot of features and characteristic attributes in SHAP of the MLP model. Each line denotes a feature. Higher eigenvalues are indicated by red dots, and lower eigenvalues are indicated by blue dots. (**E**) Comparison of the top four important features across arousal progress for non-PND (blue) and PND (red) groups. Error bars represent SEM. There is a significant statistical difference between “Log Energy Entropy” (two-way ANOVA, df = 1; F [1, 280] = 5.070, *p* = 0.0251) and “Hjorth Complexity” (two-way ANOVA, df = 1; F [1, 280] = 9.818, *p* = 0.0019). non-PND, *n* = 10; PND, *n* = 20. All data are presented as mean ± SEM. * *p* < 0.05, ** *p* < 0.01.

Model performance was compared using a core set of evaluation metrics. All algorithms were trained and validated using three-fold cross-validation combined with hold-out splits, with training-to-test ratios of 8:2, 7:3, and 6:4 (Figure S2A–C). The 8:2 split yielded the most favorable overall predictive performance (Figure S2D–F). Under this condition, receiver operating characteristic (ROC) curves (Figure S3) and confusion matrices (Figure S4) for all 12 models were generated on the test set. Under this specific split condition, the MLP classifier achieved an AUC of 0.9375, with accuracy = 0.875, precision = 1.0, recall = 0.75, and F1-score = 0.875 (Table 1; Figure 4A,B). However, because this result was derived from a single favorable split, it was not interpreted as definitive evidence of stable superiority. Accordingly, the MLP model was retained as the final classifier for downstream interpretability analyses, but should be regarded as one of the better-performing models within the current resampling framework, rather than as a definitively optimal classifier.

To further evaluate robustness, we performed repeated-resampling analyses across three train:test ratios (6:4, 7:3, and 8:2) and four random seeds (42, 300, 600, and 800). These supplementary analyses showed that classifier performance was clearly dependent on data partitioning and that no single model consistently dominated across all evaluation settings (Figure S5). Within this repeated-resampling framework, the MLP classifier remained among the better-performing models overall, although it was not uniformly superior under every split condition. Repeated-resampling evaluation of MLP itself further showed that performance was more moderate than implied by the originally highlighted single-split result and varied across split conditions (Figure S6). Accordingly, the MLP model was retained as the final classifier for downstream interpretability analyses, but should be regarded as one of the better-performing models within the current resampling framework, rather than as a definitively optimal classifier. For transparency and reproducibility, additional performance details for all 12 algorithms across different data-splitting ratios are also accessible via our web application: http://www.aibiobrain.com:8504/ (accessed on 1 April 2026).

To interpret the final classifier, the SHAP framework was used as an exploratory model-interpretation approach to estimate the relative contribution of each feature to model output within the current dataset. Based on SHAP-derived importance ranking, the top ten features were introduced stepwise into the model, and performance was evaluated as a function of feature number. Model performance reached a plateau after inclusion of the top six features (Figure 4C), indicating that these variables captured much of the discriminatory information in the current dataset.

SHAP summary bar and swarm plots (Figure 4D) visualized mean absolute SHAP values and per-instance SHAP effects, respectively, thereby ranking features by relative model importance and indicating both the direction and magnitude of their influence on model output. Higher values of Arithmetic Mean, Log Energy Entropy, Log Root Sum of Sequential Variation, and Hjorth Complexity were associated with a higher predicted probability of PND within the current model, whereas lower Beta- and Alpha-band power were associated with increased model-predicted PND probability.

We then examined the dynamic evolution of these six most important features across the full 0–100% emergence trajectory in PND and non-PND mice. Both Log Energy Entropy and Hjorth Complexity declined over the course of emergence in both groups (Figure 4E), yet remained consistently higher in PND mice than in non-PND mice, with statistically significant between-group differences. Arithmetic Mean showed a crossover pattern: before 35% emergence, values were lower in PND than in non-PND mice, whereas after 35% emergence the relationship reversed; however, no significant difference was detected when comparing the overall trajectories. Likewise, Log Root Sum of Sequential Variation, Beta-band power, and Alpha-band power (Figure 3H) did not exhibit a clear monotonic trend with increasing emergence level in either group, and no significant group differences were observed. Together, these results suggest that Log Energy Entropy and Hjorth Complexity may contribute to discrimination between PND and non-PND phenotypes at both the temporal-analysis level and within the current model, whereas the remaining features likely contribute primarily through higher-dimensional interactions rather than simple mean-level separation.

## 4. Discussion

This study established an integrated framework combining behavioral phenotyping, peri-anesthetic EEG profiling, and machine learning to identify characteristic EEG signatures and predictive models of PND vulnerability in aged mice. Behavioral analyses showed no major differences in baseline cognition across age groups, whereas anesthesia and surgery elicited marked spatial learning and contextual memory impairments exclusively in aged mice, indicating that aging-related brain frailty provides a critical substrate for PND. Using semi-supervised K-means clustering, aged surgical mice with distinct behavioral patterns were stratified into PND and non-PND subgroups. EEG analyses further demonstrated that the principal impact of aging on neuroelectric activity emerged during the arousal phase rather than during stable anesthetic maintenance. Compared with non-PND mice, PND mice displayed increased δ power and reduced α- and β-band power ratios during emergence, consistent with impaired restoration of cortical network dynamics. Finally, machine learning analysis based on emergence-phase EEG features showed that predictive performance was strongly dependent on data partitioning. Within the current repeated-resampling framework, the Multi-layer Perceptron model remained among the better-performing classifiers overall and was therefore selected for downstream interpretability analysis. Together, these findings delineate dynamic emergence-phase EEG biomarkers associated with postoperative neurocognitive vulnerability in aged mice and support the feasibility of an exploratory, explainable machine learning framework in a preclinical setting.

In this study, young and aged mice were first evaluated using the open field, Barnes maze, and contextual fear conditioning paradigms. At baseline, overall cognitive performance was comparable between age groups, apart from shorter freezing time in the contextual fear test in young mice, likely reflecting their higher baseline locomotor activity [58,59]. After anesthesia and surgery, young mice exhibited only a modest prolongation of escape latency on the second day of Barnes maze training, with preserved learning efficiency and memory retrieval. By contrast, aged mice showed pronounced postoperative cognitive impairment. Relative to aged-sham controls, aged operated mice displayed significantly prolonged escape latencies during the acquisition phase, delayed first successful entry session, fewer total successful entries, and a markedly flattened learning curve. These results indicate that aged individuals are especially vulnerable to deficits in spatial reference memory (particularly spatial learning) after general anesthesia and surgery. Consistent with our clustering-based identification of PND and non-PND subgroups, they further support the concept that aging-related cerebral frailty creates a permissive background in which perioperative stress unmasks PND, rather than perioperative exposure alone being sufficient to drive its pathogenesis.

Phenotypic identification of PND in rodent models has long impeded progress in perioperative neuroprotection research. In this study, cognitive-related behavioral profiles of aged postoperative mice were quantified using 16 behavioral fractions, and a constrained K-means clustering algorithm was applied to classify individuals as PND or non-PND. Given young mice showed stable behavior before and after surgery, postoperative behavioral data from young mice, rather than preoperative aged data, were used as the reference baseline. The constraint ensured that aged operated mice whose performance most closely resembled that of young postoperative mice clustered together, thereby segregating cognitively impaired aged individuals. Approximately two-thirds of aged surgical mice were classified as PND. Clinically, the incidence of POD after non-cardiac surgery ranges from 9% to 50%, with patients over 65 years having a 2.67-fold higher risk than younger patients and prolonged anesthesia further increasing risk [8]. To model a high-risk population, we used 18–20-month-old mice (roughly equivalent to 60–80-year-old humans [60]) and extended the anesthetic duration by 1 h beyond the conventional laparotomy PND model, yielding clustering-based PND rates at the upper end of those reported in high-risk clinical cohorts. Recently, approaches combining 3D behavioral tracking with AI-based analysis have been proposed to identify PND phenotypes from spontaneous behavior [61]; integrating such methods with our clustering strategy may enable even more precise phenotyping in future work. Importantly, this clustering result should be interpreted as a hypothesis-guided and reference-anchored stratification framework for separating aged postoperative mice into preserved/non-PND-like and vulnerable/PND-like subgroups, rather than as evidence that postoperative neurocognitive vulnerability is intrinsically binary.

Validation of the clustering results against behavioral performance confirmed their discriminative validity. In spatial memory tasks, PND mice displayed marked learning deficits during the acquisition phase of the Barnes maze, whereas memory retrieval in the probe trial remained largely preserved, indicating predominant disruption of anterograde rather than retrograde memory processes. In contextual memory tasks, PND mice exhibited significantly reduced freezing time and fewer freezing bouts, consistent with impaired contextual fear memory. As both spatial reference memory and contextual fear memory critically depend on dorsal hippocampal function [62,63], these findings support the notion that PND in aged individuals involves vulnerability of dorsal hippocampus-gated memory systems.

We next examined age- and PND-related differences in EEG dynamics during anesthetic maintenance and emergence. While young and aged mice displayed comparable oscillatory patterns under stable anesthesia, pronounced divergence emerged during arousal, with aged mice and particularly those classified as PND, showing persistent δ predominance, attenuated α and β restoration, and prolonged emergence. These findings indicate that the transition from anesthesia-induced suppression to re-established cortical-hippocampal activity represents a critical window during which aging brains exhibit heightened vulnerability. Clinically, excessive slow-wave activity and impaired recovery of fast rhythms during emergence are strongly associated with postoperative delirium in older patients, and our observations parallel these electrophysiological signatures. At the same time, this emphasis on emergence should not be taken to mean that maintenance-phase EEG is uninformative in the broader perioperative context. Recent syntheses suggest that intraoperative EEG-derived measures, particularly burst suppression and other maintenance-phase signatures, may also be associated with postoperative delirium, although the overall evidence remains heterogeneous and in some cases of low certainty [64,65]. Within the present preclinical dataset, however, emergence-phase dynamics provided greater discriminatory value for subgroup stratification and predictive modeling. The marked divergence between PND and non-PND aged mice further suggests that susceptibility is not uniformly determined by chronological age but rather by intrinsic differences in network resilience. Identifying the emergence phase as a key electrophysiological checkpoint highlights a potential therapeutic window in which stabilizing oscillatory transitions through neuromodulation or by enhancing cholinergic and noradrenergic tone may reduce PND risk.

Using EEG features extracted during emergence, machine learning analysis further suggested that peri-emergence dynamics contain predictive information relevant to postoperative neurocognitive vulnerability. Within the current repeated-resampling framework, the MLP model remained among the better-performing classifiers overall, and SHAP analysis highlighted six features with relatively strong contributions to model output. Elevated Arithmetic Mean, Log Energy Entropy, Log Root Sum of Sequential Variation, and Hjorth Complexity were strongly associated with PND, suggesting that affected mice undergo slower and less orderly neural recovery, characterized by unstable oscillations and reduced efficacy of neuronal communication [66]. Conversely, reduced α- and β-band power reflected a cortical state with diminished information-processing efficiency. These alterations collectively indicate a broader breakdown of neural stability and impaired transitions from slow-wave to higher-frequency rhythms [67], consistent with the persistent δ augmentation observed in PND mice.

While this study primarily aimed to identify emergence-phase EEG features associated with PND-like versus non-PND-like phenotypes, several limitations should be acknowledged. First, although EEG activity during both anesthesia maintenance and emergence was examined, maintenance-phase EEG was not analyzed in comparable depth because deep anesthesia is dominated by burst suppression, which is strongly influenced by anesthetic dose and may therefore obscure intrinsic network-level differences. In our cohort, aged mice showed a non-significant trend toward higher burst suppression ratios during maintenance. Consistent with this, a recent cardiac surgery trial reported that intraoperative titration to reduce burst suppression did not significantly lower the incidence of POD [19]. Together, these findings suggest that maintenance-phase EEG alone may have limited discriminatory value in the present dataset, whereas emergence dynamics provided stronger neurophysiological separation. At the same time, the machine learning representation summarized the emergence period into one feature vector per animal, which reduced cross-subject misalignment caused by variable emergence duration but inevitably compressed part of the temporal recovery structure. Future studies should incorporate more explicit trajectory-level descriptors, such as slopes along normalized emergence progression, time-resolved feature curves, or sequence-based models. Second, PND is an umbrella construct that includes both POD and longer-term postoperative cognitive decline across different temporal windows [68], and it encompasses not only cognitive impairment but also alterations in emotion, attention, and executive function [67,69]. In the present study, our behavioral fractions were mainly designed to probe learning and memory. Anxiety- and depression-like behaviors were not systematically assessed, in part due to the lack of validated perioperative behavioral batteries in rodents, and this represents an important area for future refinement. Third, sex-related factors may modulate both behavior and EEG signatures. Prior work has documented sex differences in spatial reference memory strategies [70], sex-specific circuit recruitment during contextual fear conditioning [71,72], and estrous cycle-dependent modulation of hippocampal spine density and theta–gamma oscillations [73,74,75]. To minimize hormonal and circuit-level confounding, we restricted the current analysis to male mice. Therefore, the present findings should be interpreted as specific to aged male mice, and future studies will need to explicitly evaluate female cohorts and sex as a biological variable. Finally, the EEG features and prediction model identified here have not been validated in human perioperative EEG datasets. Rodent EEG provides a useful preclinical tool for studying anesthesia-related brain-state transitions, and several large-scale neurophysiological principles may be partially conserved across species; however, rodent and human EEG are not directly equivalent due to differences in anatomical scale, cortical geometry, recording context, and detailed spectral organization. Therefore, prospective validation across human cohorts, anesthesia protocols, and heterogeneous patient populations will be necessary before any clinical applicability can be inferred.

## 5. Conclusions

In conclusion, this preclinical study strengthens the concept that EEG during emergence phase carries prognostic information relevant to PND in aged subjects. To our knowledge, we first established an individual-level phenotyping strategy for PND in aged surgical mice, based on standardized neurocognitive behavioral assessments and semi-supervised clustering. We then delineated distinct emergence-phase EEG trajectories in aged mice with and without PND and showed that an MLP-based exploratory classifier trained on these signals could distinguish PND-vulnerable mice from a preserved/non-PND-like reference group within the current preclinical dataset. Together, these findings define emergence-phase EEG signatures linked to postoperative neurocognitive vulnerability in aged mice and provide a mechanistic and analytic framework for future translational studies. Validation in human perioperative EEG cohorts will be required to determine whether these signatures generalize across clinical settings.

## Figures and Tables

**Table 1:** Classification performance of different models in PND prediction with the training set and test set ratios of 8:2.

	Performance	Accuracy	Precision	Recall	F1	AUC
Models						
LR		0.75	0.67	1.00	0.80	0.81
CatBoost		0.75	0.75	0.75	0.75	0.88
DT		0.75	0.75	0.75	0.75	0.75
RF		0.88	0.80	1.00	0.89	1.00
AdaBoost		0.75	0.75	0.75	0.75	0.78
ET		1.00	1.00	1.00	1.00	1.00
SVM		0.75	1.00	0.5	0.67	0.88
NB		0.75	0.67	1.00	0.80	0.81
KNN		0.63	0.67	0.50	0.57	0.66
MLP		0.88	1.00	0.75	0.86	0.94
XGBoost		0.75	0.75	0.75	0.75	0.88
LightGBM		0.50	0.50	1.00	0.67	0.50

AUC, area under the receiver operating characteristic curve; LR, Logistic Regression; DT, Deci-sion Tree; RF, Random Forest; ET, Extra Trees; SVM, Support Vector Machine; NB, Gaussian Naive Bayes; KNN, K-Nearest Neighbors; MLP, Multi-layer Perceptron; XGBoost, eXtreme Gradient Boosting; LightGBM, Light Gradient Boosting Machine.

## Data Availability

The data presented in this study are available on request from the corresponding author. The data are not publicly available because they have not been deposited in a public repository.

## Supplementary Materials

The following supporting information can be downloaded at: https://www.mdpi.com/article/10.3390/diagnostics16081186/s1, Figure S1: EEG feature differences between non-PND and PND mice during anesthetic emergence. Figure S2: Performance score index of 12 artificial intelligence models under different division ratios. Figure S3: Confusion matrix graph of 12 AI models in test sets under an 8:2 division ratio. Figure S4: ROC curves of 12 AI models in test sets under an 8:2 division ratio. Figure S5: Repeated-resampling AUC comparison of the principal competing classifiers across three train:test ratios: 6:4, 7:3, and 8:2. Figure S6: Repeated-resampling test performance of the Multi-layer Perceptron (MLP) classifier across three train:test ratios: 6:4, 7:3, and 8:2. Figure S7: Correlation heatmap of the 33 predefined EEG features. Figure S8: Stability of SHAP-derived top features across repeated random seeds. Table S1: Comparison of alternative unconstrained clustering methods. Table S2: Definitions of EEG features. Table S3: Repeated-resampling AUC values of the principal competing classifiers across different train:test ratios and random seeds. Table S4. Exploratory pairwise comparisons of repeated-resampling AUC values between the Multi-layer Perceptron and the principal competing classifiers within each train:test ratio.
