# Supplementary material for: The Perioperative Neurocognitive Disorder Prediction Based on AI-Assisted EEG Dynamic Features in Anesthetized Mice
Source: Diagnostics (Basel). 2026 Apr 16;16(8):1186. doi: 10.3390/diagnostics16081186 (PMC13114885; doi:10.3390/diagnostics16081186)
Supplement: Supplementary file 1 [file diagnostics-16-01186-s001.zip › diagnostics-4162822-supplementary.pdf]

# Supplementary Information

## The Perioperative Neurocognitive Disorder Prediction Based on AI-assisted EEG Dynamic Features in Anesthetized Mice

Xinyang Li 1,†, Hui Wang 2,3,4,†, Qingyuan Miao 1,†, Rui Zhou 1, Mengfan He 1, Hanxi Wan 1, Yuxin Zhang 1, Qian Zhang 1, Zhouxiang Li 1, Qianqian Wu 1, Zhi Tao 1, Xinwei Huang 1, Enduo Feng 1, Qiong Liu 1, Yinggang Zheng 1,*, Guangchao Zhao 2,3,4,* and Lize Xiong 1,*

[1] Shanghai Key Laboratory of Anesthesiology and Brain Functional Modulation, Translational Research Institute of Brain and Brain-Like Intelligence, Clinical Research Centre for Anesthesiology and Periopera-tive Medicine, Department of Anesthesiology and Perioperative Medicine, Shanghai Fourth People's Hos-pital, School of Medicine, Tongji University, Shanghai 200434, China

[2] Department of Anesthesiology and Perioperative Medicine, Xijing Hospital, The Fourth Military Medical University, Xi'an 710032, China

[3] Key Laboratory of Anesthesiology, Ministry of Education of China, Xi'an 710032, China

[4] Innovation Research Institute, Xijing Hospital, The Fourth Military Medical University, Xi'an 710032, China

* Correspondence: ygzheng@tongji.edu.cn (Y.Z.); gczhao0518@hotmail.com (G.Z.); lizexiong@tongji.edu.cn or mzkxlz@126.com (L.X.)

† These authors contributed equally to this work.

# Materials and methods

### *Alternative unconstrained clustering comparison*

To examine whether the subgroup structure depended strongly on the specific clustering framework, we performed an additional sensitivity analysis using several unconstrained clustering methods, including K-means, spectral clustering, hierarchical clustering, DBSCAN, and Gaussian mixture modeling (GMM) (**Table S1**). This analysis was performed without semi-supervised constraints and was intended to compare clustering behavior, balance, and interpretability across methods, rather than to reproduce the final constrained subgroup assignment used in the main analysis.

Among the tested methods, spectral clustering and GMM produced numerically better internal clustering indices, but both resulted in highly imbalanced partitions (36/2 and 34/4, respectively), indicating trivial solutions dominated by a large majority cluster and a very small outlier cluster. DBSCAN failed to identify valid clusters under the tested parameter setting, labeling all 38 samples as noise. Hierarchical clustering generated a more balanced partition (21/17), but showed poorer internal validity than K-means. K-means also generated a balanced partition (21/17) and provided the most reasonable compromise among cluster balance, internal validity, and biological interpretability, which is especially relevant in small-sample, high-dimensional biomedical data where clustering validity should not be judged solely by a single internal index [1]. On this basis, and because the final subgrouping in the main analysis was hypothesis-guided and reference-anchored, we retained the semi-supervised/constrained K-means framework for downstream analyses.

***Clustering stability analysis across random seeds***

To evaluate the reproducibility of subgroup assignment, constrained K-means clustering was repeated across 200 random seeds while enforcing the same prior constraints. Pairwise agreement between clustering results was quantified using the Adjusted Rand Index (ARI), a standard measure of agreement between clustering partitions [2], and the resulting 1−ARI distance matrix was subjected to hierarchical clustering to identify stable seed groups. Seven stable groups were identified using a within-group ARI threshold of >0.7. The largest stable group contained 75 seeds (37.5%), with a mean within-group ARI of 0.847 ± 0.123. A representative seed from this dominant stable group (seed 33) yielded a balanced partition (20 PND/18 non-PND), with all eight prior samples assigned to the same cluster. These results indicate the presence of a reproducible dominant clustering pattern within the constrained K-means framework.

***Machine learning prediction model development***

***Machine learning model***

**LR:** A linear model for binary classification that predicts the probability of a sample belonging to a class using the logistic function.

**CatBoost:** A gradient boosting framework developed by Yandex that effectively handles categorical features without the need for explicit encoding. It introduces ordered target statistics to prevent overfitting and uses symmetric trees to ensure stable performance, making it ideal for classification and regression tasks with mixed data types.

**DT:** A tree-based model that recursively splits the feature space based on decision rules.

**RF:** An ensemble method that constructs multiple decision trees and aggregates their predictions via majority voting or averaging, improving robustness and reducing overfitting.

**AdaBoost:** A machine learning algorithm that belongs to the family of boosting algorithms. It works by converting weak learners into strong learners through iterative reweighting of training instances, giving higher weights to incorrectly classified instances and lower weights to correctly classified ones.

**ET:** A tree-based machine learning model similar to random forests but with a different approach to splitting nodes. Instead of searching for the best split point at each node, it uses random splits, which can lead to faster training and sometimes better performance, especially in high-dimensional spaces.

**SVM:** A classifier that finds the optimal hyperplane to separate classes, maximizing the margin between them, with kernel tricks for non-linear boundaries.

**NB:** A probabilistic machine learning model based on Bayes' theorem. It assumes that the features are conditionally independent given the class, which is often not true in practice. It is particularly useful for classification tasks with continuous features, where each feature follows a Gaussian distribution.

**KNN:** A non-parametric algorithm that classifies a sample based on the majority class of its k-nearest neighbors, relying on distance metrics like Euclidean distance.

**MLP:** A basic artificial neural network (ANN) with an input layer, one or more

hidden layers, and an output layer. It uses non-linear activation functions to model complex relationships between inputs and outputs, making it suitable for a wide range of classification and regression tasks.

**XGBoost:** A gradient boosting framework that sequentially builds decision trees, optimizing a loss function to achieve high performance and scalability.

**LightGBM:** A gradient boosting framework that uses tree-based learning algorithms. It is developed by Microsoft and is designed to be more efficient than other boosting algorithms, especially with large-scale and high-dimensional data.

***Repeated-resampling robustness analysis***

To further assess the robustness of model performance, repeated-resampling analyses were performed under three train:test ratios (6:4, 7:3, and 8:2) using four random seeds (42, 300, 600, and 800). For each split condition, the principal competing classifiers were re-evaluated using the same preprocessing and training workflow described in the main Methods. Repeated-resampling AUC values were then summarized to compare relative classifier behavior across different data-partition settings. These repeated-resampling AUC summaries are provided in **Table S3**. In addition, repeated-resampling test performance of the Multi-layer Perceptron (MLP) model was summarized using accuracy, AUC, F1-score, precision, and recall.

Repeated-resampling analyses showed that classifier performance was clearly dependent on the train:test split, and no single model consistently dominated across all evaluation settings (**Figure S5**). Exploratory pairwise comparisons between MLP and the principal competing classifiers within each split condition are summarized in **Table**

**S4**. Among the five principal competing classifiers, the MLP model remained among the better-performing models overall, although it was not uniformly superior under every split condition. Repeated-resampling evaluation of the MLP classifier further showed that performance was more moderate than implied by the originally highlighted single-split result and varied across split conditions (**Figure S6**). In particular, test-set AUC was lower under the 6:4 and 7:3 settings and higher under the 8:2 setting, indicating that predictive performance was sensitive to data partitioning. These findings support a more cautious interpretation of the current classifier as an exploratory, proof-of-concept framework rather than a stable or definitive predictive model.

***Feature correlation analysis***

To assess redundancy within the predefined EEG feature space, we examined the pairwise correlation structure of the 33 predefined EEG features (**Figure S7**). Multiple feature groups showed strong positive or negative correlations, indicating substantial redundancy within the full feature set. These results support interpreting the reduced-feature analysis in **Figure 4C** as a post hoc compact interpretability analysis rather than a pretraining dimensionality-reduction procedure.

***SHAP feature-ranking stability across random seeds***

To assess the stability of SHAP-based feature importance, we compared the top SHAP-ranked features of the final MLP classifier across four random seeds (42, 300, 600, and 800) (**Figure S8**). Several features recurred across multiple seeds, indicating partial consistency of the SHAP-derived importance structure. However, the exact ranking and composition of the top features varied across runs, indicating that SHAP-

based feature attribution in the current small-sample setting should be interpreted cautiously as exploratory rather than definitive.

## Supplementary Figure

### Figure S1

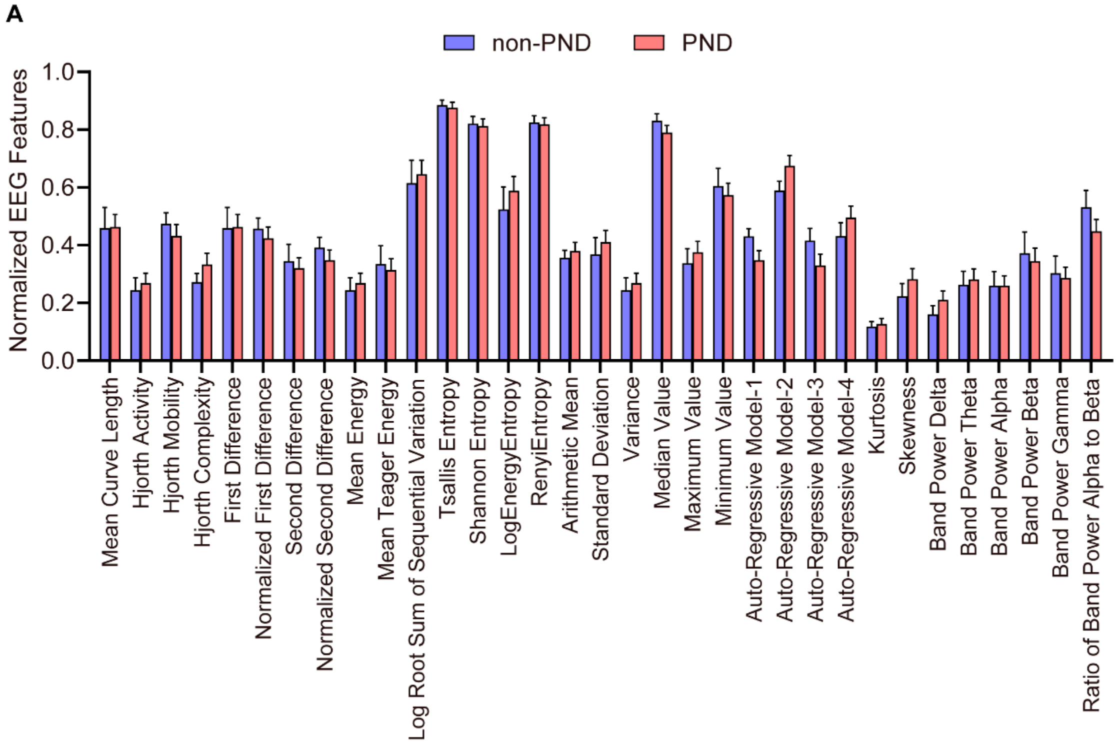


**EEG feature differences between non-PND and PND mice during anesthetic emergence.** (**A**) Comparison of 33 standardized EEG features between non-PND and PND mice during anesthetic emergence (Unpaired t test, df=28). non-PND, n = 10; PND, n = 20. Data are represented as mean ± SEM.

## Figure S2

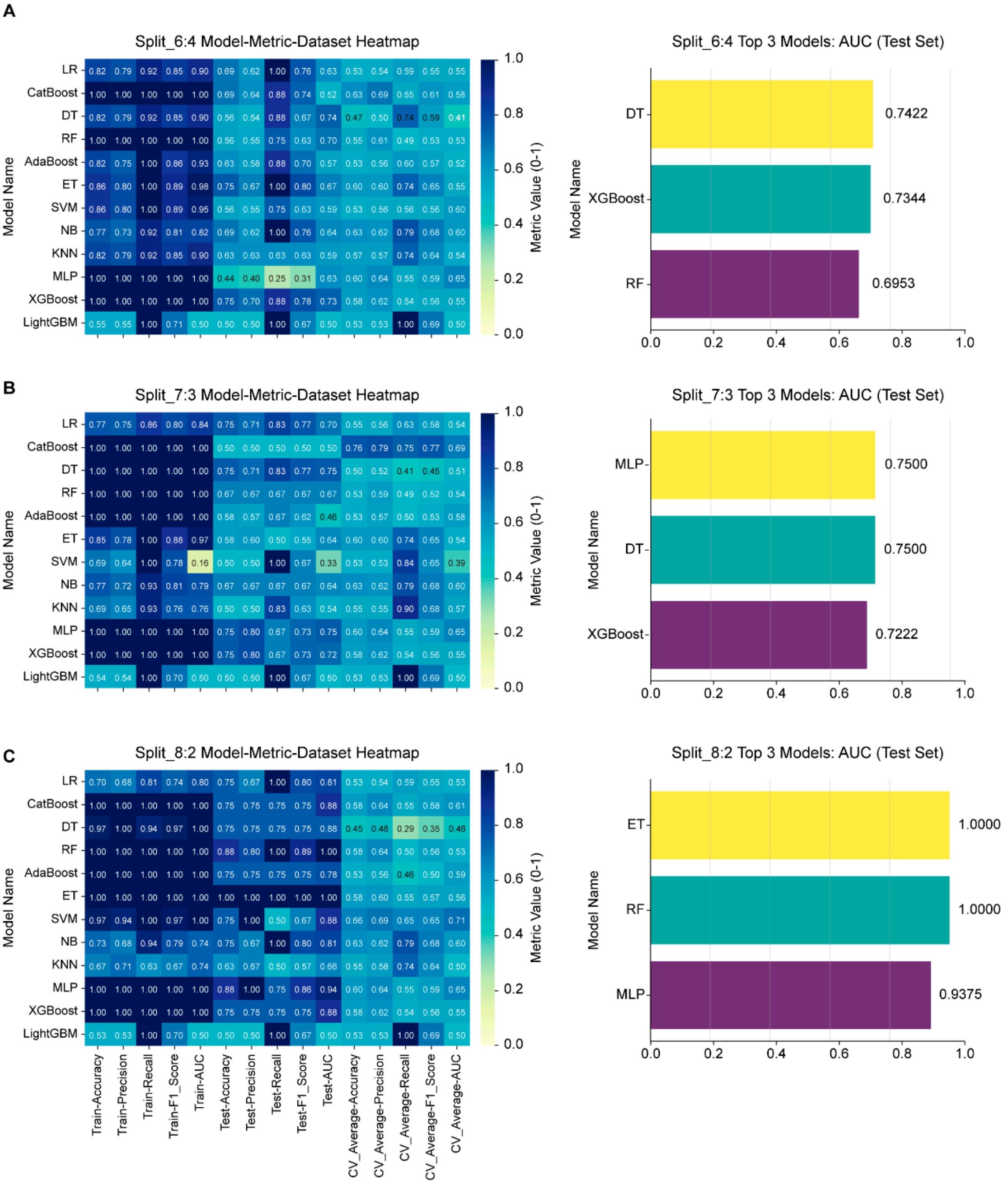


**Performance score index of 12 artificial intelligence models under different division ratios.** (**A**) Performance score heat map (left) and the scores of the top three AUCs (right) of 12 artificial intelligence models under a 6:4 ratio. (**B**) Performance score heat map (left) and the scores of the top three AUCs (right) of 12 artificial intelligence models under a 7:3 ratio. (**C**) Performance score heat map (left) and the scores of the top three AUCs (right) of 12 machine learning models under an 8:2 ratio.

## Figure S3

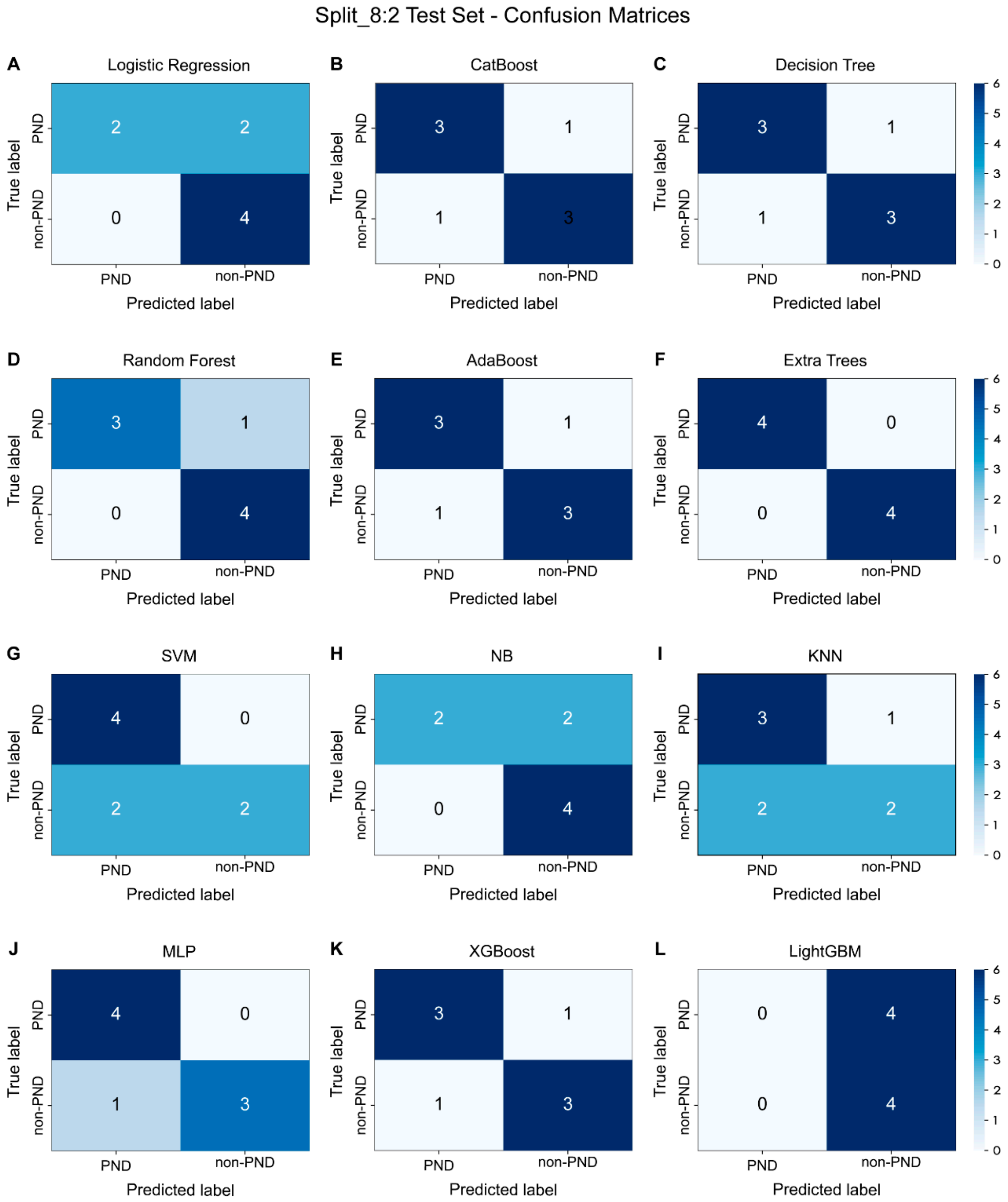


**Confusion matrix graph of 12 AI models in test sets under an 8:2 division ratio.** Confusion matrix graph of Logistic Regression (**A**), CatBoost (**B**), Decision Tree (**C**), Random Forest (**D**), AdaBoost (**E**), Extra Trees (**F**), SVM (**G**), NB (**H**), KNN (**I**), MLP (**J**), XGBoost (**K**), and LightGBM (**L**) models in test sets under an 8:2 division ratio.

## Figure S4

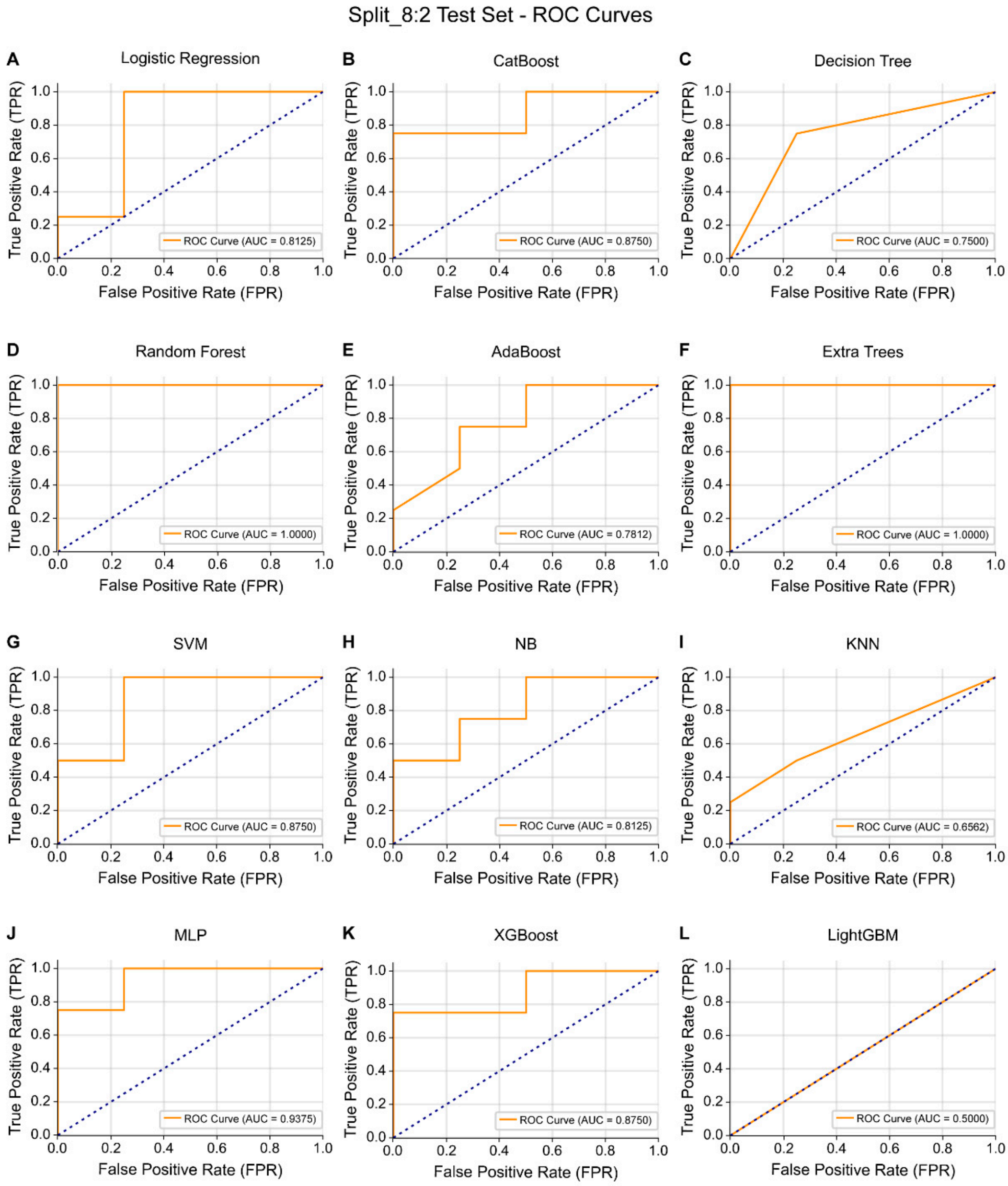


**ROC curves of 12 AI models in test sets under an 8:2 division ratio.** ROC curves of Logistic Regression (**A**), CatBoost (**B**), Decision Tree (**C**), Random Forest (**D**), AdaBoost (**E**), Extra Trees (**F**), SVM (**G**), NB (**H**), KNN (**I**), MLP (**J**), XGBoost (**K**), and LightGBM (**L**) models in test sets under an 8:2 division ratio.

**Figure S5**

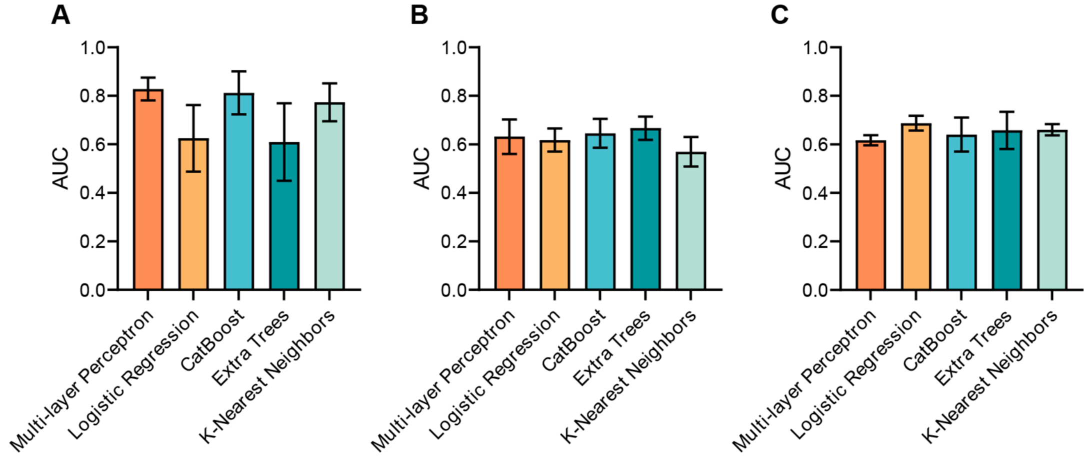


**Repeated-resampling AUC comparison of the principal competing classifiers across three train:test ratios: 6:4 (A), 7:3 (B), and 8:2 (C).** Five representative models were compared, including Multi-layer Perceptron, Logistic Regression, CatBoost, Extra Trees, and K-Nearest Neighbors. Bars represent mean AUC ± SEM across four random seeds (42, 300, 600, and 800). This analysis was used to compare the relative performance of the major competing classifiers under different evaluation conditions.

**Figure S6**

A

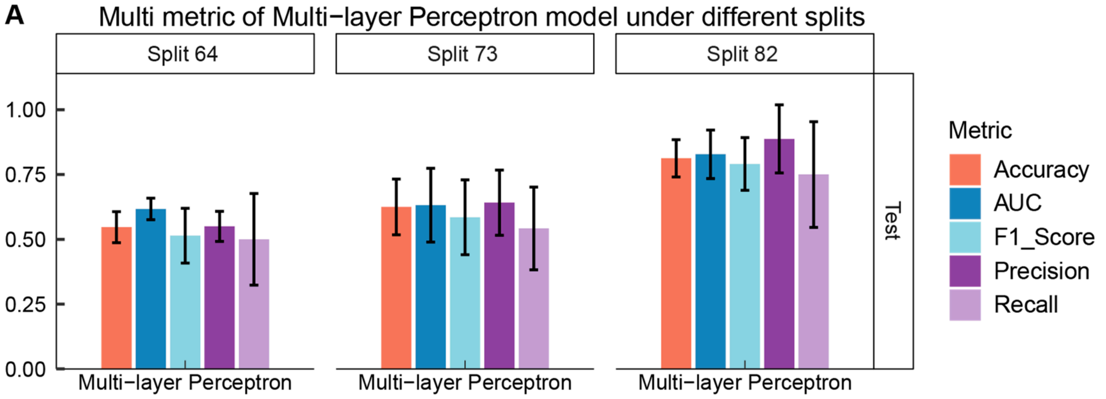


**Repeated-resampling test performance of the Multi-layer Perceptron (MLP) classifier across three train:test ratios: 6:4, 7:3, and 8:2.** Accuracy, AUC, F1-score, precision, and recall are shown as mean ± SEM across four random seeds (42, 300, 600, and 800). Mean test AUC of MLP was **0.617** under 6:4, **0.632** under 7:3, and **0.828** under 8:2. This figure illustrates the dependence of MLP performance on data partitioning under repeated resampling.

## Figure S7

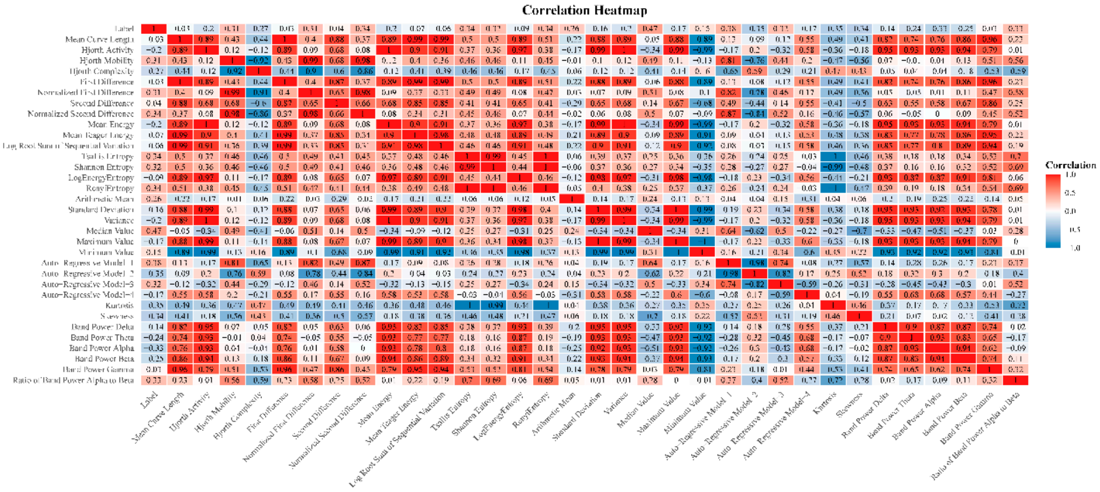


**Correlation heatmap of the 33 predefined EEG features.** Pairwise correlations among the 33 predefined EEG features are shown to illustrate redundancy within the predefined feature space.

## Figure S8

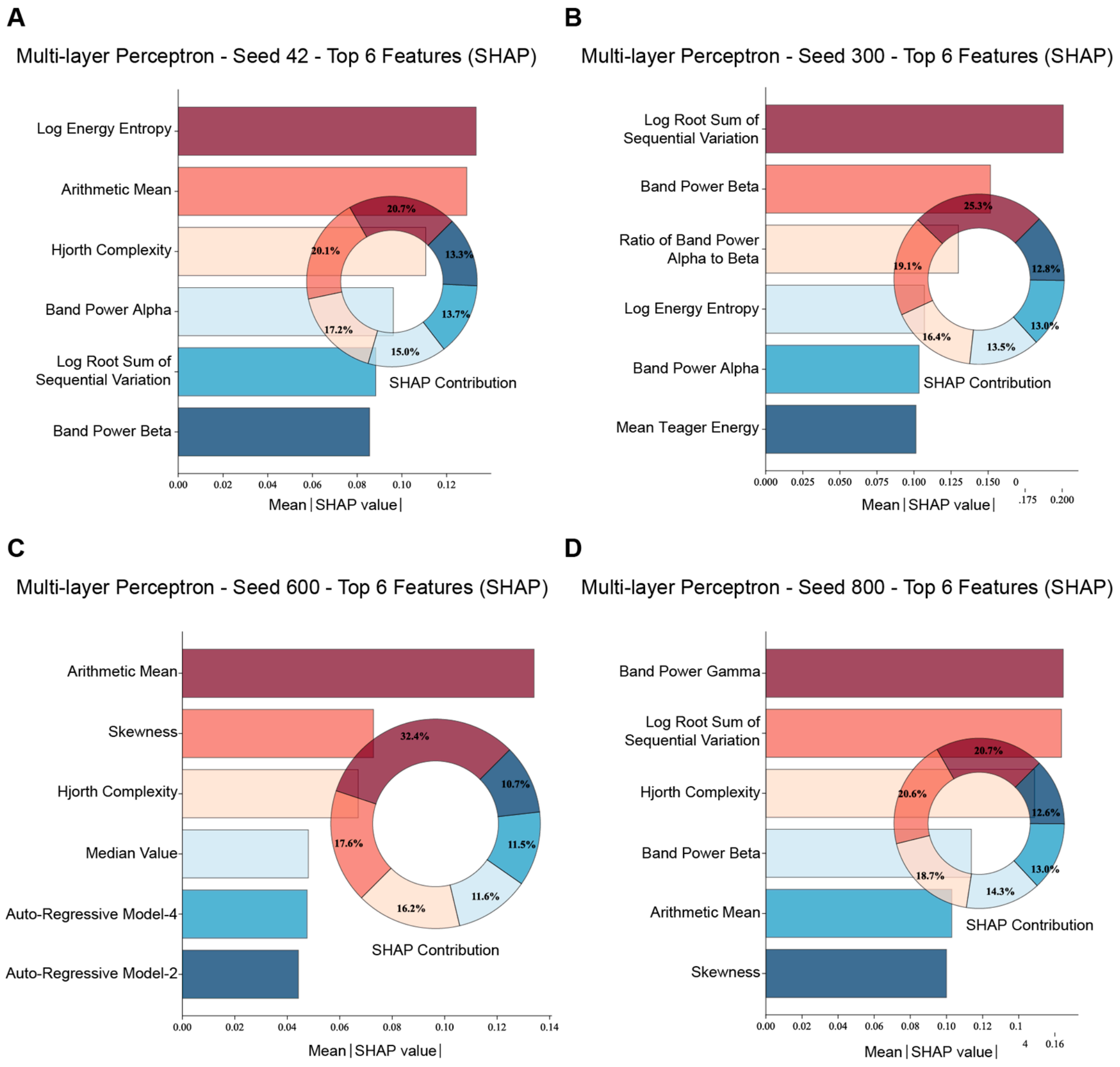


**E** Feature recurrence across seeds

| | | | | | |
|---|---|---|---|---|---|
| Arithmetic Mean | 4/4 | Band Power Alpha | 2/4 | Auto-Regressive Model-4 | 1/4 |
| Hjorth Complexity | 3/4 | Skewness | 2/4 | Auto-Regressive Model-2 | 1/4 |
| Log Root Sum of Sequential Variation | 3/4 | Ratio of Band Power Alpha to Beta | 1/4 | Band Power Gamma | 1/4 |
| Band Power Beta | 3/4 | Mean Teager Energy | 1/4 | | |
| Log Energy Entropy | 2/4 | Median Value | 1/4 | | |

**Stability of SHAP-derived top features across repeated random seeds.** (**A–D**) Top six SHAP-ranked features of the final MLP classifier obtained under four random seeds (42, 300, 600, and 800). (**E**) Frequency of occurrence of the top six SHAP-ranked features across the four random seeds.

# Supplementary Table

## Table S1. Comparison of alternative unconstrained clustering methods.

| Method | All 8 prior samples in the same cluster | Cluster size distribution | Silhouette coefficient | Davies–Bouldin index |
|---|---|---|---|---|
| K-means | No | 21/17 | 0.161 | 1.900 |
| Spectral clustering | Yes | 36/2 | 0.224 | 0.947 |
| Hierarchical clustering | No | 21/17 | 0.134 | 2.041 |
| DBSCAN | Yes | all 38 labeled as noise | N/A | N/A |
| Gaussian mixture model (GMM) | Yes | 34/4 | 0.222 | 1.484 |

Alternative clustering methods were evaluated in an unconstrained setting for sensitivity analysis of subgroup structure. “All 8 prior samples in the same cluster” indicates whether the eight reference samples were assigned to a common cluster without imposing any semi-supervised constraint. DBSCAN labeled all 38 samples as noise under the tested parameter setting and therefore did not yield valid clustering partitions; accordingly, silhouette coefficient and Davies–Bouldin index were not applicable. Although spectral clustering and GMM yielded numerically better internal clustering indices, both produced highly imbalanced partitions, whereas K-means provided a more balanced and biologically interpretable solution.

**Table S2. Definitions of EEG Features**

| EEG Feature | Definition |
|---|---|
| Power (Frequency-domain) | Delta power band (0.5-4 Hz), Theta power band (4-8 Hz), Alpha power band (8-12 Hz), Beta power band (12-30 Hz), and Gamma power band (more than 30 Hz), reflecting the distribution of signal energy across different frequency ranges. The Alpha-to-Beta ratio indicates the relative dominance of these frequency bands. |
| Mean Teager energy | The instantaneous energy accounts for both amplitude and frequency. |
| Mean energy | The average of squared amplitude values indicates overall signal intensity. |
| Mean curve length | Average of the first-order difference absolute values of the EEG signal, capturing signal smoothness. |
| Log Root Sum of Sequential Variation | The logarithm of the square root of the sum of squared differences quantifies the variability in the signal's sequential changes. |
| Minimum | The smallest amplitude value in the EEG signal over a given time window indicates the lower bound of signal intensity. |
| Median | The middle value of the EEG signal amplitudes when sorted. Robust to outliers and provides a central tendency measure. |
| Maximum | The largest amplitude value in the EEG signal over a given time window indicates the upper bound of signal intensity. |
| Arithmetic mean | The average value of the EEG signal amplitudes represents the signal's central tendency. |
| Standard deviation | A measure of the degree of dispersion of the EEG signal distribution |
| Variance | The square of the standard deviation. Quantifies the spread of the EEG signal's amplitude distribution. |
| Skewness | The asymmetry of the amplitude distribution indicates whether the signal has more extreme values on one side. |
| Kurtosis | The peakedness of the amplitude distribution reflects the presence of extreme values. |
| First difference | The difference between consecutive samples measures the signal change rate. |
| Normalized first difference | A normalized measure of self-similarity based on the first difference. |
| Second difference | The difference of the first difference captures higher-order changes. |
| Normalized Second difference | A normalized measure of self-similarity based on the second difference. |
| Auto-regressive (AR) Model 1-4 coefficients | The EEG signal itself is used as the linear process of a regression variable to estimate the power spectrum, and the order of the model is selected as 4 |
| Hjorth activity | The signal's variance represents total power or amplitude variation. |
| Hjorth mobility | The mean frequency, estimated as the ratio of the standard deviation of the first derivative to the signal's standard deviation. |

| | |
|---|---|
| Hjorth complexity | The signal's bandwidth, computed by comparing the mobility of the first derivative to that of the signal itself, indicates waveform complexity. |
| Shannon entropy | The expected information content reflects signal unpredictability. |
| Tsallis entropy | A measure of signal disorder, generalizing Shannon entropy. |
| Renyi entropy | An approximation of spectral complexity based on the signal's probability distribution. |
| Log Energy entropy | A measurement of the complexity of the EEG signal, quantifying the complexity of the EEG signal based on the logarithm of the signal's energy and reflecting the distribution of energy across the signal. |

**Table S3. Repeated-resampling AUC values of the principal competing classifiers across different train:test ratios and random seeds**

| Resampling | Multi-layer Perceptron | Logistic Regression | CatBoost | Extra Trees | K-Nearest Neighbors |
|---|---|---|---|---|---|
| 8:2_seed 42 | 0.94 | 0.94 | 0.94 | 0.94 | 0.91 |
| 8:2_seed 300 | 0.88 | 0.75 | 0.94 | 0.81 | 0.88 |
| 8:2_seed 600 | 0.75 | 0.50 | 0.56 | 0.44 | 0.75 |
| 8:2_seed 800 | 0.75 | 0.31 | 0.81 | 0.25 | 0.56 |
| 7:3_seed 42 | 0.78 | 0.69 | 0.72 | 0.64 | 0.44 |
| 7:3_seed 300 | 0.69 | 0.69 | 0.67 | 0.81 | 0.74 |
| 7:3_seed 600 | 0.61 | 0.58 | 0.47 | 0.64 | 0.56 |
| 7:3_seed 800 | 0.44 | 0.50 | 0.72 | 0.58 | 0.54 |
| 6:4_seed 42 | 0.64 | 0.63 | 0.66 | 0.75 | 0.61 |
| 6:4_seed 300 | 0.61 | 0.77 | 0.73 | 0.67 | 0.64 |
| 6:4_seed 600 | 0.66 | 0.70 | 0.44 | 0.44 | 0.67 |
| 6:4_seed 800 | 0.56 | 0.66 | 0.73 | 0.77 | 0.72 |

**Table S4. Exploratory pairwise comparisons of repeated-resampling AUC values between the Multi-layer Perceptron and the principal competing classifiers within each train:test ratio**

| Split ratio | Comparison | Mean AUC (MLP) | Mean AUC (Comparator) | Wilcoxon matched-pairs *p* |
|---|---|---|---|---|
| 8:2 | MLP vs LR | 0.83 | 0.63 | 0.25 |
| 8:2 | MLP vs CatBoost | 0.83 | 0.81 | >0.9999 |
| 8:2 | MLP vs ET | 0.83 | 0.61 | 0.25 |
| 8:2 | MLP vs KNN | 0.83 | 0.77 | 0.50 |
| 7:3 | MLP vs LR | 0.63 | 0.62 | 0.75 |
| 7:3 | MLP vs CatBoost | 0.63 | 0.65 | 0.88 |
| 7:3 | MLP vs ET | 0.63 | 0.67 | 0.75 |
| 7:3 | MLP vs KNN | 0.63 | 0.57 | 0.88 |
| 6:4 | MLP vs LR | 0.62 | 0.69 | 0.25 |
| 6:4 | MLP vs CatBoost | 0.62 | 0.64 | 0.88 |
| 6:4 | MLP vs ET | 0.62 | 0.66 | 0.88 |
| 6:4 | MLP vs KNN | 0.62 | 0.66 | 0.50 |

## References


1. Handl, J.; Knowles, J.; Kell, D.B. Computational cluster validation in post-genomic data analysis. Bioinformatics **2005**, 21, 3201-3212, doi:10.1093/bioinformatics/bti517.
2. Hubert, L.; Arabie, P. Comparing partitions. Journal of Classification **1985**, 2, 193-218, doi:10.1007/BF01908075.
